# Teachers' Perceptions of the Pil-smart Model: A Semlj Analysis of its Influence on Science and Environmental Literacy in Early Childhood Education

**DOI:** 10.12688/f1000research.181997.1

**Published:** 2026-06-30

**Authors:** Nita Priyanti, Chandra Apriyansyah, Tedy Andrian

**Affiliations:** 1 Early Childhood Education, Universitas Panca Sakti Bekasi, Bekasi, West Java, 17414, Indonesia

**Keywords:** Pil-Smart learning model, science literacy, environmental literacy, early childhood education, SEM

## Abstract

**Background:**

The development of science literacy and environmental literacy in early childhood education is important to build critical thinking skills and ecological awareness from an early age. However, the implementation of a learning model that integrates the two literacies is still limited. In addition, research on teachers’ perceptions of innovative learning models oriented to these two literacies is also still minimal. In fact, teachers’ perceptions play an important role in determining the success of learning implementation. Therefore, this study aims to analyze teachers’ perceptions of the PIL-SMART learning model and its influence on science literacy and environmental literacy.

**Method:**

This study uses a quantitative approach with a survey design. A total of 200 PAUD teachers were selected through purposive sampling. Data were collected using questionnaires that included three constructs: PIL-SMART, science literacy, and environmental literacy. The analysis was performed using Structural Equation Modeling (SEM) with the help of Jamovi (SEMLj) to test the relationship between latent variables and model feasibility.

**Results:**

Model shows excellent match (χ ^2^ = 402; p = 0.485; RMSEA = 0.002; CFI = 0.991; TLI = 0.990). Teachers’ perception of PIL-SMART had a significant effect on science literacy (β = −0.125; p = 0.043). However, there was no significant effect on environmental literacy (β = 0.046; p = 0.091). Science literacy has a significant effect on environmental literacy (β = −0.100; p = 0.010).

**Conclusion:**

PIL-SMART is effective in improving science literacy, but it has no direct effect on environmental literacy. Science literacy plays a role as a liaison in the development of environmental literacy. Strengthening exploration-based learning and hands-on experience is needed to support both literacy in an integrated manner.

## 1. Research background

The development of the 21 ^st^ century brings major changes in various aspects of life, including the field of education. An increasingly complex world, characterized by technological advances, environmental changes, and global challenges such as the climate crisis and the degradation of natural resources, demands the birth of a generation capable of thinking critically, adaptively, and responsibly ( Moffit, 2023; Şentürk, 2017). In this context, science literacy and environmental literacy are two fundamental competencies that need to be developed systematically from an early age ( Agal & Seal, 2025). Science literacy is not only concerned with mastery of scientific concepts or facts, but also includes the ability of children to observe, question, investigate, and draw conclusions based on concrete experiences ( Chaesar & Andayani, 2024). Meanwhile, environmental literacy emphasizes understanding and caring behavior for the environment ( Isa et al., 2021). Both are interrelated in forming scientific thinking skills as well as children’s ecological awareness from an early age.

However, learning practices in early childhood education still face various challenges Learning activities tend to be dominated by a teacher-centered approach, where teachers are the main source of information and children play the role of passive recipients. In addition, learning is often presented separately between developmental areas, such as science, art, religion, and language, so that children’s learning experiences become fragmented and less meaningful ( Amira & Kadir, 2025; Ramulumo, 2025). Inquiry-based approaches and project-based learning that encourage exploration, observation, and contextual problem-solving have also not been optimized in learning practices. This condition has an impact on the lack of development of science literacy and environmental literacy as a whole and the lack of linkage between learning and children’s real experiences.

In this situation, teachers play a central role in determining the quality of learning in the classroom, as they act as designers, implementers, and evaluators of the learning process. Teachers’ perceptions of a learning model greatly affect the consistency and effectiveness of its implementation in daily practice ( Chandra et al., 2023; Feng & Zhang, 2023; Williams et al., 2023). When teachers view the learning model as relevant, easy to understand, and in accordance with the needs and characteristics of children, the implementation tends to be more optimal, directed, and sustainable ( Irawan et al., 2024; Moffit, 2023). On the other hand, a less positive perception can have an impact on the low quality of learning implementation. Therefore, the analysis of the effectiveness of a learning model needs to consider the teacher’s perspective comprehensively, not only focusing on the child’s learning outcomes, but also on the teacher’s experience and meaning in the implementation process.

In response to these needs, the PIL-SMART (Project, Inquiry, Science, Mathematics, Art, Religion, and Technology) model was developed as an integrative and interdisciplinary approach in early childhood learning. This model combines project-based learning and inquiry in a single framework that connects different aspects of child development. Through exploration and investigation activities that are integrated with the context of daily life, children are encouraged to actively build knowledge while developing concern for the environment ( Chaesar & Andayani, 2024; Lestari et al., 2025; Liberali et al., 2025). Thus, this model has the potential to develop science literacy and environmental literacy in a holistic and meaningful way.

Although studies have shown that inquiry-based and project-based approaches can improve children’s learning outcomes and environmental awareness, most studies still focus on children’s achievement directly. Research that examines the relationship between variables structurally based on teachers’ perceptions is still relatively limited In addition, the integration of science literacy and environmental literacy in one interdisciplinary learning model in the context of early childhood education has not been widely explored, and the use of the Jamovi-based Structural Equation Modeling (SEMLj) approach in this context is also still rarely done.

Based on these gaps, this study aims to analyze the influence of the PIL-SMART model on science literacy and environmental literacy based on the perception of PAUD teachers. Theoretically, this model is seen as able to improve both types of literacy through the integration of project-based learning, exploration of the real environment, and a scientific approach that encourages children to actively observe, question, try, and reflect on learning experiences. The SEMLj approach is used to test the relationships between latent constructs simultaneously so that a more comprehensive understanding of the influence patterns that occur is obtained. The novelty of this research lies in the development of an interdisciplinary integrative model in early childhood education, testing two types of literacy in one structural framework, and the use of teachers’ perspectives in the Indonesian context. This research is expected to make a theoretical, methodological, and practical contribution to strengthening early childhood education learning oriented to science literacy and environmental sustainability.

## 2. Literature review

### 2.1 PIL-SMART learning method

Project Inquiry Learning Science, Mathematics, Art, Religion, and Technology (PIL-SMART) is an integrated learning model based on constructivism, inquiry, and project-based learning theory. In line with Piaget’s view, children build their knowledge through direct experience ( Lev et al., 2020; Siraj-Blatchford, 2016). Therefore, project-based learning provides children with the opportunity to be actively involved and construct meaningful understanding through their own activities ( Odell & Kennedy, 2020; Strawhacker et al., 2020). Dewey and Bruner emphasized that the inquiry process through questioning, investigating, and discovering activities has an important role in developing children’s scientific thinking skills ( Nimmo & Park, 2009; Wight et al., 2016). In the PIL-SMART model, this process is realized by integrating science, mathematics, and art concepts into learning activities that are close to the child’s experience, so that learning becomes more contextual and easy to understand.

Vygotsky emphasized the importance of social interaction and scaffolding in the learning process. In the context of PIL-SMART, this is reflected in the role of teachers as facilitators and the existence of cooperation between children during the implementation of the project ( Daniele et al., 2025; Ramulumo, 2025). The integration of religious values in this model is in line with the view of character education that emphasizes the cultivation of moral values through real experience. Meanwhile, the use of technology helps children develop digital literacy as a means to explore and document learning outcomes. Thus, PIL-SMART can be understood as a holistic learning model that supports the development of early childhood cognitive, social, emotional, moral, and creative abilities in a balanced manner.

### 2.2 Science literacy

Science literacy is understood as the ability of individuals to understand scientific concepts and processes and apply them in explaining phenomena, interpreting evidence, and making responsible decisions in daily life ( Aunillah, 2024; Rai et al., 2025). Science literacy is also interpreted as the active involvement of individuals in scientific issues based on scientific reasoning and evidence. The National Research Council emphasizes the importance of integrating conceptual knowledge and scientific practice, such as observing, questioning, investigating, and interpreting data ( Chandra et al., 2023; Hong et al., 2019). In addition, science literacy includes critical thinking and problem-solving skills in social and technological contexts ( Liana et al., 2024; Thiel, 2025).

In the context of early childhood education, science literacy can be developed from an early age through hands-on experience that is appropriate to the child’s developmental stage ( Fitriani, 2024). Science literacy is also seen as a means to shape social attitudes, values, and responsibilities, including concern for the environment ( Agal & Seal, 2025). Thus, science literacy is not only oriented towards mastering concepts, but also on developing process skills, scientific attitudes, and social awareness holistically.

H1: Teachers’ perceptions of the PIL-SMART learning model have a significant effect on science literacy in early childhood education.

### 2.3 Environmental literacy

Environmental literacy is understood as the ability of individuals to understand environmental systems and problems and show attitudes and behaviors that are responsible for preserving nature. Environmental literacy encompasses a combination of ecological knowledge, awareness, and skills in making decisions related to the environment ( Isa et al., 2021). In addition, environmental literacy emphasizes the integration of knowledge, thinking skills, caring attitudes, and pro-environmental behaviors as the core of its formation ( Masykuroh et al., 2024).

Environmental literacy is part of education for sustainable development that emphasizes the formation of value and responsibility towards nature from an early age ( Guerrero Fernández et al., 2022). Its development takes place effectively through direct experience and real interaction with the surrounding environment. In early childhood, environmental literacy is built through habituation, play activities, and strengthening attitudes that respect nature in daily life ( Feng & Zhang, 2023; J. Li et al., 2025). Therefore, environmental literacy focuses not only on conceptual understanding, but also on the formation of sustainable attitudes and behaviors as a whole.

H2: Teachers’ perceptions of the PIL-SMART learning model have a significant effect on environmental literacy in early childhood education.

H3: Science literacy has a significant effect on environmental literacy in early childhood education.

**Figure 1.**
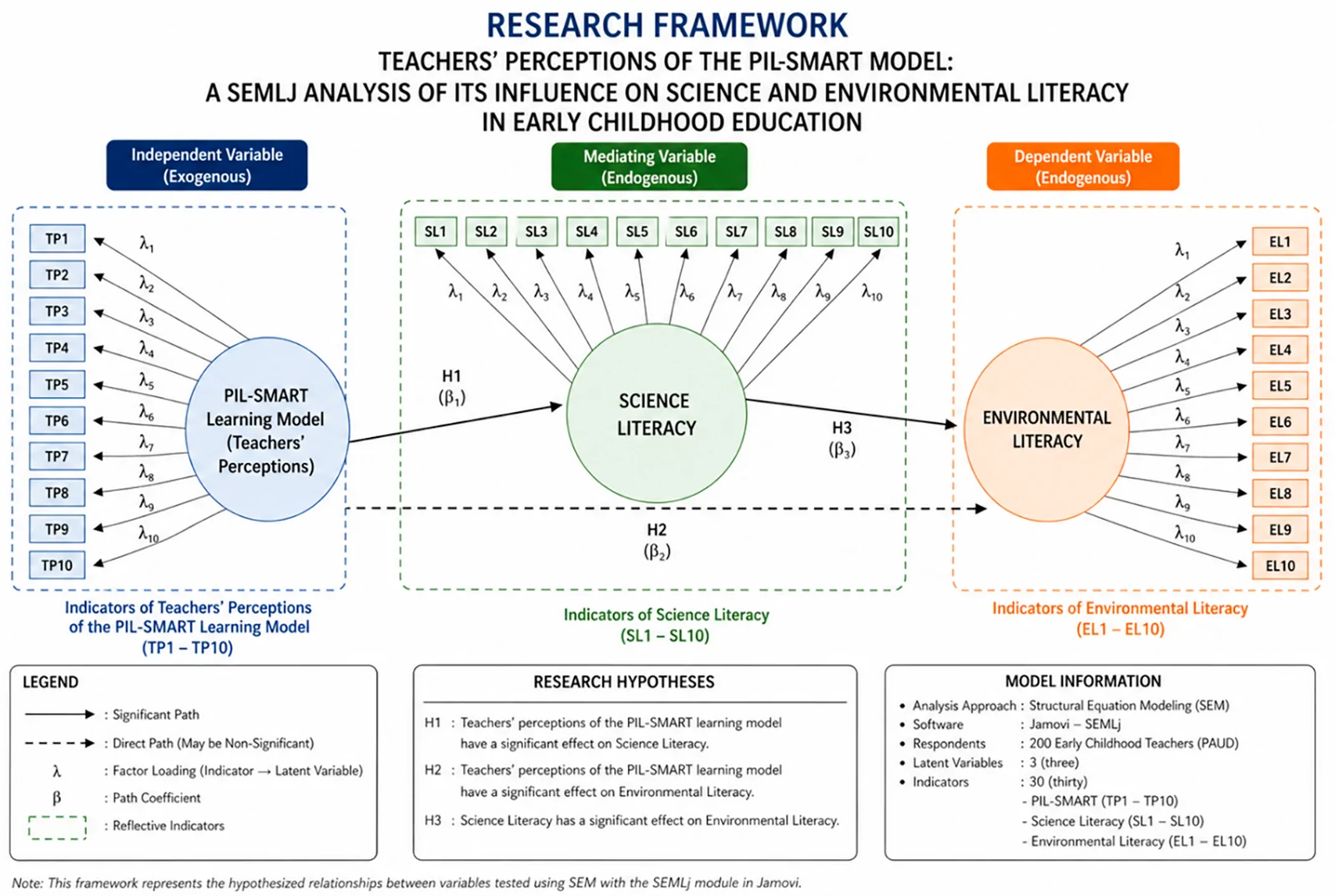
Research framework.

The conceptual framework of this study illustrates the structural relationships among the PIL-SMART learning model, science literacy, and environmental literacy in early childhood education, as presented in Figure 1. The framework explains that teachers’ perceptions of the implementation of the PIL-SMART model are assumed to influence the development of children’s science literacy and environmental literacy. In addition, science literacy is also hypothesized to contribute to environmental literacy development. The framework was developed based on theories of inquiry learning, project-based learning, and literacy development in early childhood education. Therefore, Figure 1 serves as the basis for testing the relationships among variables through the Structural Equation Modeling (SEM) analysis used in this study.

## 3. Research methods

### 3.1 Samples and procedures

This study uses a quantitative approach with a survey design to analyze teachers’ perceptions of the implementation of the Pil-Smart learning model and its influence on science literacy and environmental literacy in early childhood education. The quantitative approach was chosen because this study aims to test the relationship between variables using measurable and objective statistical analysis. The sampling technique used is purposive sampling, which is the selection of respondents based on certain criteria that are relevant to the purpose of the research. The respondent criteria in this study are teachers who actively teach in early childhood education institutions and have experience in implementing science-based and environment-based learning activities.

The number of samples in this study is 200 early childhood education teachers from 125 PAUD institutions. This amount is considered adequate for Structural Equation Modeling analysis because it meets the minimum sample size recommendations in SEM analysis. Data collection was carried out using questionnaires that were distributed directly or through online media to respondents. Before data collection was carried out, respondents were given an explanation of the purpose of the research and the guarantee of data confidentiality provided. All respondents agreed to participate in the study voluntarily.

The research procedure is carried out through several main stages. The first stage is the preparation of research instruments based on literature reviews on innovative learning models, science literacy, and environmental literacy in early childhood. The second stage is to test the validity of the instrument’s content through discussions with early childhood education experts and educational evaluation experts. The third stage is the distribution of questionnaires to the study respondents. The last stage is data processing and analysis using the Structural Equation Modeling method to test the relationship between research variables.

Prior to data collection, ethical approval for this study was obtained from the Ethical Clearance Commission, Research Institutions and Community Service (LPPM), Panca Sakti University Bekasi, Indonesia, under Ethical Clearance Certificate No. 001/LPPM.ECC/PSUB/V/2026. All participants were informed about the objectives of the study, the voluntary nature of participation, anonymity, and confidentiality of their responses. Informed consent was obtained verbally from all participants before the questionnaire administration. Verbal informed consent was selected instead of written consent because the study involved minimal risk, did not collect sensitive personal data, and was conducted using questionnaire-based participation to ensure respondents’ comfort and practical accessibility during data collection.

### 3.2 Respondent profile

The profile of the respondents in this study describes the characteristics of early childhood education teachers who participated in the study. The characteristics of the respondents were analyzed based on several aspects, namely gender, age, education level, and length of teaching experience. This information is important to provide an overview of the background of the respondents involved in the study. In addition, respondent profile data also helps in understanding the context of the implementation of the Pil-Smart learning model in classroom learning practices.

Based on the results of data analysis, most of the respondents in this study were female teachers who worked in early childhood education institutions. This is in accordance with the general conditions in PAUD institutions where most of the educators are women. In terms of age, the majority of respondents are in the productive age range between 25 to 40 years. The age range shows that most respondents have sufficient professional experience in early childhood learning activities.

In terms of education level, most respondents have a bachelor’s education background in the field of early childhood education or relevant education. This shows that respondents have adequate academic competence in understanding the concept of learning and child development. In addition, some respondents have also participated in various professional trainings related to the development of innovative learning methods. The training experience helps teachers in implementing a more creative and contextual approach to learning.

Judging from teaching experience, the majority of respondents have between five and ten years of teaching experience. The experience provides a deeper understanding of the characteristics of early childhood learning. Teachers who have sufficient teaching experience tend to be more able to adapt various learning models in teaching and learning activities. Therefore, the respondents in this study are considered to have relevant experience in providing an assessment of the Pil-Smart learning model.

### 3.3 Measurement analysis using SEMLj

The data analysis in this study was carried out using Structural Equation Modeling (SEM) with the help of Jamovi software through the SEMLj module. The SEM method is used because it is able to analyze the complex relationships between several latent variables simultaneously. In addition, SEM also allows researchers to test measurement models and structural models simultaneously. This analysis was used to determine the relationship between teachers’ perception of the Pil-Smart learning model and science literacy and environmental literacy in early childhood.

The research model consists of three latent constructs, namely Pil-Smart, science literacy, and environmental literacy. The Pil-Smart construct is measured using ten indicators that describe teachers’ perceptions of the implementation of the learning model. The construct of science literacy is also measured using ten indicators that describe children’s ability to understand simple science concepts. Meanwhile, the construct of environmental literacy is measured using ten indicators related to children’s awareness and behavior towards the surrounding environment. All indicators are measured using a five-point Likert scale.

The SEM analysis stage is carried out through several steps. The first step is to test the measurement model to determine the validity of the construct through the analysis of the loading factor on each indicator. The second step is the evaluation of the feasibility of the model using various goodness of fit indices such as Chi-square, RMSEA, SRMR, CFI, and TLI. The third step is to test the structural model to find out the relationships between latent variables using the values of the path coefficient and the level of significance.

In addition, the analysis also includes testing the coefficient of determination (R ^2^) to find out the extent to which the dependent variables can be explained by the research model. The results of the SEM analysis were then interpreted to explain the relationship between the Pil-Smart learning model and science literacy and environmental literacy in early childhood. The entire analysis process is carried out using the latest version of Jamovi software equipped with the SEMLj module. Thus, the analysis method used is able to provide comprehensive results in explaining the relationship between research variables.

## 4. Research results

### 4.1 Model fit evaluation

Table 1 presents the results of the model fit evaluation obtained from the Structural Equation Modeling (SEM) analysis.

**Table 1.** Model fit evaluation.

Label	X ^2^	df	p
A Letter to the Editor	402	402	.485
Baseline Model	482	435	.059
Scaled User	407	402	.418
Scaled Baseline	460	435	.197

*Note*: lavaan- > lav_object_post_check(): some estimated lv variances are negativelavaan- > lav_object_post_check(): some estimated lv variances are negative.

Based on Table 1, the chi-square value obtained was χ ^2^ = 402 with df = 402 and a significance value of p = 0.485. Since the significance value was greater than 0.05, the proposed model did not significantly differ from the observed data. This finding indicates that the research model had an acceptable level of fit with the empirical data. In addition, the baseline and scaled model results also supported the adequacy of the SEM model. Therefore, the proposed structural model was considered appropriate for further analysis.

Table 2 and Table 3 present the goodness-of-fit indices used to evaluate the suitability of the SEM model.

**Table 2.** Goodness-of-fit indices.

			95% Confidence Intervals		
Type	SRMR	RMSEA	Lower	Upper	RMSEA p
Classical	0.074	0.002	0.000	0.025	1.000
Robust	0.068	0.024	0.000	0.039	.999
Scaled	0.068	0.008	0.000	0.027	1.000

**Table 3.** Comparative model fit indices.

	Models	Scaled	Robust
Comparative Fit Index (CFI)	0.991	0.789	0.549
Tucker-Lewis Index (TLI)	0.990	0.772	0.512
Bentler-Bonett Non-normed Fit Index (NNFI)	0.990	0.772	0.512
Relative Noncentrality Index (RNI)	0.991	0.789	0.549
Bentler-Bonett Normed Fit Index (NFI)	0.165	0.115	
Bollen’s Relative Fit Index (RFI)	0.097	0.042	
Bollen’s Incremental Fit Index (IFI)	0.995	0.909	
Parsimony Normed Fit Index (PNFI)	0.153	0.106	

The RMSEA value shown in Table 2 was 0.002, which was below the recommended threshold of 0.08, indicating a very small approximation error. Furthermore, the SRMR value was 0.074, which also satisfied the acceptable criterion of less than 0.08. As shown in Table 3, the Comparative Fit Index (CFI) value was 0.991 and the Tucker-Lewis Index (TLI) value was 0.990. Both indices exceeded the recommended value of 0.90, indicating that the model had an excellent fit with the observed data. Overall, these findings confirmed that the proposed SEM model was feasible and suitable for structural analysis.

### 4.2 Measurement model

Table 4 presents the factor loading results for the measurement model used in this study.

**Table 4.** Measurement model results.

				95% Confidence intervals				
Latent	Observed	Estimate	OR	Lower	Upper	β	z	p
Squirrel	TP1	1.0000	0.000	1.0000	1.0000	0.21962		
	TP2	−0.1140	0.503	−1.0993	0.8713	−0.02504	−0.2268	.821
	TP3	0.7265	0.490	−0.2332	1.6863	0.15957	1.4837	.138
	TP4	−0.5210	0.508	−1.5162	0.4743	−0.11442	−1.0260	.305
	TP5	0.0242	0.478	−0.9119	0.9603	0.00532	0.0507	.960
	TP6	0.7288	0.497	−0.2459	1.7036	0.16007	1.4655	.143
	TP7	−0.5632	0.481	−1.5063	0.3798	−0.12370	−1.1705	.242
	TP8	−0.5318	0.409	−1.3340	0.2704	−0.11680	−1.2994	.194
	TP9	−0.4733	0.488	−1.4301	0.4834	−0.10396	−0.9697	.332
	TP10	0.8879	0.632	−0.3513	2.1271	0.19501	1.4044	.160
Science_Literacy	SL1	1.0000	0.000	1.0000	1.0000	0.55848		
	SL2	0.4830	0.209	0.0730	0.8929	0.26973	2.3090	.021
	SL3	0.2438	0.191	−0.1314	0.6191	0.13618	1.2736	.203
	SL4	−0.3266	0.190	−0.6995	0.0462	−0.18242	−1.7172	.086
	SL5	0.4366	0.196	0.0519	0.8214	0.24386	2.2244	.026
	SL6	0.0717	0.179	−0.2794	0.4229	0.04007	0.4005	.689
	SL7	0.0755	0.184	−0.2853	0.4363	0.04214	0.4099	.682
	SL8	0.2692	0.178	−0.0791	0.6176	0.15036	1.5149	.130
	SL9	−0.2938	0.209	−0.7034	0.1158	−0.16407	−1.4057	.160
	SL10	−0.4649	0.191	−0.8387	−0.0911	−0.25965	−2.4378	.015
Environmental_Literacy	EL1	1.0000	0.000	1.0000	1.0000			
	EL2	0.5898	0.306	−0.0103	1.1899		1.9264	.054
	EL3	−0.6043	0.374	−1.3381	0.1296		−1.6139	.107
	EL4	0.2814	0.250	−0.2078	0.7706		1.1276	.259
	EL5	−0.3329	0.258	−0.8383	0.1725		−1.2911	.197
	EL6	0.9146	0.455	0.0219	1.8073		2.0081	.045
	EL7	1.4839	0.689	0.1339	2.8338		2.1543	.031
	EL8	0.4154	0.298	−0.1687	0.9995		1.3939	.163
	EL9	0.0326	0.229	−0.4167	0.4819		0.1421	.887
	EL10	0.2298	0.251	−0.2614	0.7210		0.9170	.359

The findings showed that the indicators contributed differently to their respective latent constructs. In the Pil-Smart construct, the TP10 indicator demonstrated the highest loading factor value of 0.8879, indicating that it had the strongest contribution in representing the construct. Several additional indicators also showed positive loading values, suggesting that they adequately represented the dimensions of the Pil-Smart learning model. These findings indicate that the indicators used in this construct were capable of explaining teachers’ perceptions of the Pil-Smart model. Therefore, the Pil-Smart construct was considered sufficiently represented by the selected indicators.

As shown in Table 4, several indicators in the science literacy and environmental literacy constructs demonstrated statistically significant loading values. In the science literacy construct, SL2 (β = 0.483, p = 0.021) and SL5 (β = 0.436, p = 0.026) significantly contributed to the construct. Meanwhile, in the environmental literacy construct, EL6 (β = 0.9146, p = 0.045) and EL7 (β = 1.4839, p = 0.031) showed strong loading values and significant contributions. These findings indicate that the indicators effectively represented children’s science literacy and environmental literacy abilities. Overall, the results confirmed that the indicators adequately represented the latent constructs included in the SEM model.

### 4.3 Structural model

**Figure 2.**
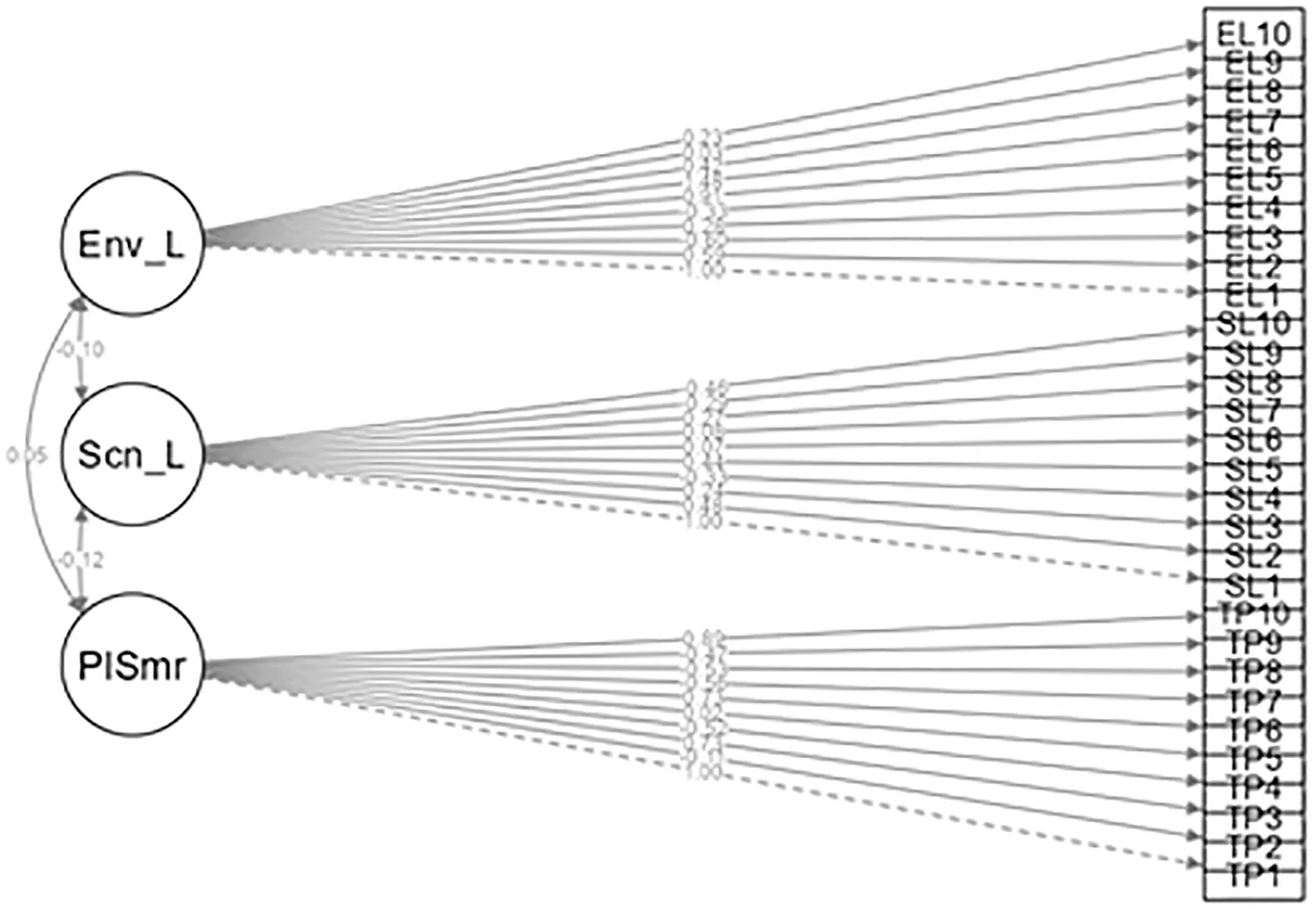
Structural model of pil-smart, science literacy, and environmental literacy.

Figure 2 illustrates the structural relationships among Pil-Smart, science literacy, and environmental literacy in the SEM model.

The results showed that Pil-Smart had a significant relationship with science literacy, with a path coefficient value of β = −0.125 and p = 0.043. This finding indicates that teachers’ perceptions of the Pil-Smart learning model were associated with the development of children’s science literacy. The significant relationship suggests that innovative learning approaches may support children’s understanding of science concepts and exploration activities. Therefore, the Pil-Smart model has the potential to contribute positively to science literacy development in early childhood education. The structural relationship shown in Figure 2 confirms the importance of learning innovation in improving children’s literacy skills.

However, the relationship between Pil-Smart and environmental literacy was not statistically significant, as indicated by the path coefficient value of β = 0.0469 with p = 0.091. This finding suggests that the Pil-Smart learning model did not directly influence children’s environmental literacy development. In contrast, science literacy demonstrated a significant relationship with environmental literacy, with a path coefficient value of β = −0.1004 and p = 0.010. This result indicates that children who demonstrated better science literacy also tended to show stronger environmental literacy abilities. Overall, the structural model shown in Figure 2 provides an empirical explanation of the relationships among the variables included in this study.

## 5. Discussion

The results of this study show that the Pil-Smart learning model has a significant influence on science literacy in early childhood education. These findings show that an exploratory and hands-on experience learning approach is able to improve children’s understanding of basic science concepts. In early childhood education, the learning process involving concrete activities is very important because children learn through interaction with the surrounding environment. The Pil-Smart model provides children with the opportunity to observe and explore directly. Through these activities, children can develop curiosity and scientific thinking skills from an early age. Therefore, this learning model can be an effective strategy in supporting the development of science literacy in children.

The findings of this study are in line with various previous studies that show that experiential learning can improve children’s science literacy skills. Early childhood tends to understand science concepts through exploratory activities such as observing, trying, and experimenting ( Cutrer-Párraga et al., 2020; Moffit, 2023; Quro & Choiriyah, 2022). These activities help children understand the causal relationships that occur in natural phenomena. Learning that emphasizes hands-on experience can also increase children’s involvement in the learning process ( Brandt et al., 2025; Smolkin & Donovan, 2015). In addition, exploration activities allow children to develop critical thinking skills from an early age. Thus, an exploratory learning approach has great potential in improving science literacy ( Singh, 2022; Thiel, 2025).

The results of this study also show that teachers’ perceptions of learning models have an important role in the early childhood learning process. Teachers who have a positive perception of innovative learning models tend to be more active in implementing creative learning strategies ( Aunillah, 2024; Şentürk, 2017). Teachers’ perceptions can influence how they design learning activities that actively engage children. In early childhood education, teachers play the role of facilitators who help children find knowledge through learning experiences ( Hong et al., 2019). Therefore, teachers’ understanding of the learning model is an important factor in the successful implementation of the model. These findings show that teacher competency development is indispensable in the implementation of innovative learning models.

However, the results of this study show that the Pil-Smart model does not have a significant direct influence on environmental literacy. These findings suggest that the development of environmental literacy in early childhood is likely influenced by a variety of other factors outside of the learning model used ( Clark et al., 2020; Salleh & Omar, 2025). Environmental literacy is not only related to knowledge, but also related to attitudes and behaviors towards the environment. Forming an attitude of caring for the environment usually requires a longer and more continuous learning process. In addition, children’s experiences in daily life can also affect the development of their environmental literacy ( Suharti et al., 2020). Therefore, environmental learning needs to be carried out consistently in various learning activities.

The relationship between science literacy and environmental literacy found in this study shows that there is a relationship between the two concepts. Understanding science concepts can help children understand environmental phenomena that occur around them ( An et al., 2019; Rafiq-uz-Zaman, 2025). For example, an understanding of water, soil, and plants can help children understand the importance of taking care of the environment. This knowledge can help children understand the relationship between humans and nature. By understanding the basic concepts of science, children can develop awareness of the importance of protecting the environment. Therefore, science literacy can be an important basis for the development of environmental literacy in early childhood.

These findings suggest that the integration of science and environmental learning is essential in early childhood education. Learning that combines science concepts with activities related to the environment can help children understand the relationship between humans and nature more comprehensively. Activities such as planting plants, observing animals, or keeping the environment clean can help children understand these concepts concretely. Contextual learning activities can also increase children’s involvement in the learning process. In addition, these activities can also help children develop an attitude of caring for the environment from an early age ( Britsch, 2017; Robertson & Moran, 2019). Therefore, the integration of science and environmental learning needs to be strengthened in learning activities.

The Pil-Smart model provides opportunities for teachers to develop more creative and contextual learning activities. The learning approach used in this model encourages children to be active in the learning process. Children not only receive information passively but also engage in exploratory activities. These activities help children build knowledge through learning experiences ( Daniele et al., 2025). In addition, an interactive learning approach can also increase children’s motivation to learn. Thus, the Pil-Smart model can be an effective learning strategy in early childhood education.

The findings of this study also provide practical implications for early childhood education teachers. Teachers need to design learning activities that are able to integrate various aspects of learning holistically. Learning that combines science concepts with activities related to the environment can help children understand natural phenomena better. In addition, learning activities also need to be designed to suit the characteristics of early childhood development ( Chen & Delaney, 2025; L. Li & Tan, 2015). Teachers can use a variety of activities such as simple experiments and environmental observation activities. Thus, learning can be more interesting and meaningful for children.

In the context of early childhood education, literacy development is one of the important aspects of the learning process. Literacy is not only related to the ability to read and write but also includes the ability to understand the world around children ( Daniele et al., 2025; Ramulumo, 2025). Science literacy and environmental literacy are an important part of developing these skills. Literacy development from an early age can help children develop critical thinking skills ( Ly-Hoang, 2023b, 2023a). In addition, literacy also helps children understand the relationship between humans and the environment. Therefore, early childhood education needs to pay greater attention to literacy development.

This research also contributes to the development of academic studies on innovative learning models in early childhood education. This study shows that a systematically designed learning model can have an impact on children’s literacy development. In addition, this study also shows that teacher perception is an important factor in the successful implementation of the learning model. These findings reinforce the importance of teacher competency development in implementing innovative learning approaches ( Marufah et al., 2025; Tariq, 2025). Thus, this research contributes to enriching the study of learning innovations in early childhood education.

Although this research makes an important contribution, there are some limitations that need to be considered. This study only involved a number of respondents from certain early childhood education institutions. Therefore, the results of the study may not be able to be generalized widely. In addition, this study uses a survey approach that relies on respondents’ perceptions ( Ly-Hoang, 2023b; Preston, 2018). Teachers’ perceptions can be influenced by their personal experiences as well as the context of their work environment. Therefore, further research can involve a wider sample to obtain more representative results.

Further research can also develop a more integrated learning model between science and the environment. Such integration can help children understand the relationship between humans and nature more comprehensively ( Daniele et al., 2025; Ly-Hoang, 2023a). In addition, further research can also use a mixed method approach to gain a deeper understanding of the implementation of learning models. The approach allows researchers to combine quantitative and qualitative data ( Abraham, 2021; Ly-Hoang, 2023b). Thus, the results of the research can provide a more comprehensive picture of the learning process. Follow-up research can also examine the influence of other factors that affect children’s environmental literacy.

Overall, this study shows that the Pil-Smart learning model has the potential to improve science literacy in early childhood education. Although its influence on environmental literacy is not significant, the relationship between science literacy and environmental literacy shows a connection between the two concepts. This shows that science learning can be the basis for the development of environmental awareness in children. Therefore, the integration of science and environmental learning needs to continue to be developed in early childhood education. The development of innovative learning models can help create a more meaningful learning experience for children. Thus, this research makes an important contribution to the development of learning strategies in early childhood education.

## 6. Conclusion

This study aims to analyze teachers’ perceptions of the Pil-Smart learning model and its influence on science literacy and environmental literacy in early childhood education. The results of the analysis using Structural Equation Modeling show that the research model has a good degree of conformity with empirical data. The findings of the study show that the Pil-Smart learning model has a significant influence on science literacy in early childhood. This shows that an exploratory and experiential learning approach can help children understand science concepts more effectively. The Pil-Smart model provides children with the opportunity to learn through activities that involve environmental observation and exploration. Therefore, this learning model has the potential to improve science literacy skills in early childhood.

However, the results of the study show that the Pil-Smart model does not have a significant direct influence on environmental literacy. These findings suggest that the development of environmental literacy in early childhood is likely influenced by a variety of other factors outside of the learning model used. Environmental literacy is not only related to understanding concepts, but also related to attitudes and behaviors towards the environment. Forming an attitude of caring for the environment usually requires continuous experience in daily life. Therefore, the development of environmental literacy needs to be carried out through the integration of more contextual and sustainable learning activities. This shows that learning that integrates science and environmental activities needs to be continuously developed in early childhood education.

In addition, this study also shows a significant relationship between science literacy and environmental literacy. These findings show that understanding science concepts can help children understand environmental phenomena around them. By understanding the concept of science, children can develop an awareness of the relationship between humans and the environment. This shows that science literacy can be an important basis for the development of environmental literacy in early childhood. Therefore, learning that integrates science and environmental concepts can help children understand the world around them more comprehensively. Overall, this research contributes to the development of innovative learning models that can support the development of children’s literacy in early childhood education.

## Ethical considerations

Ethical approval for this study was obtained from the Ethical Clearance Commission, Research Institutions and Community Service (LPPM), Panca Sakti University Bekasi, Indonesia, under Ethical Clearance Certificate No. 001/LPPM.ECC/PSUB/V/2026. The study complied with institutional ethical standards for research involving human participants. Informed consent was obtained from all participants prior to data collection.

## Data Availability

Zenodo: Analysis Result Data for Research Entitled Teachers’ Perceptions of The PIL-SMART Model: A SEMLJ Analysis Of Its Influence on Science and Environmental Literacy in Early Childhood Education https://doi.org/10.5281/zenodo.20305973 ( Priyanti et al., 2026). This project contains the following extended data:
1.
data_kuesioner_SEM_PLS_200_responden.xlsx 2. Structural Equation Modelling.omv 3. Structural Equation Modelling.pdf
