# Teachers' Perceptions of the PIL-SMART Model: A Semlj Analysis of its Influence on Science and Environmental Literacy in Early Childhood Education

**DOI:** 10.12688/f1000research.181997.2

**Published:** 2026-08-19

**Authors:** Nita Priyanti, Chandra Apriyansyah, Tedy Andrian

**Affiliations:** 1 Early Childhood Education, Universitas Panca Sakti Bekasi, Bekasi, West Java, 17414, Indonesia

**Keywords:** Pil-Smart learning model, science literacy, environmental literacy, early childhood education, SEM

## Abstract

**Background:**

Science and environmental literacy are foundations for children’s scientific thinking and ecological awareness. However, integrated learning approaches connecting these competencies remain limited in early childhood education. This study examined associations among teachers’ perceptions of Project–Inquiry Learning integrating Science, Mathematics, Art, Religion, and Technology (PIL-SMART), teacher-rated science literacy, and teacher-rated environmental literacy.

**Methods:**

A cross-sectional survey involved 200 teachers from 125 early childhood education institutions in Bekasi City, West Java, Indonesia. Participants were selected through purposive sampling. Data were collected using a five-point Likert-scale questionnaire measuring three constructs. Reliability analysis, Confirmatory Factor Analysis, and Structural Equation Modeling were conducted using Jamovi with the SEMLj module. Following measurement-model evaluation, 28 indicators were retained: ten for PIL-SMART, nine for science literacy, and nine for environmental literacy.

**Results:**

The model demonstrated fit, χ ^2^ (347) = 408, p = 0.014, CFI = 0.980, TLI = 0.978, RMSEA = 0.030 (95% CI: 0.014–0.041), and SRMR = 0.049. PIL-SMART perceptions were positively associated with science literacy (β = 0.744, p < 0.001) and environmental literacy (β = 0.266, p = 0.006). Science literacy was positively associated with environmental literacy (β = 0.517, p < 0.001). The indirect association through science literacy was significant (β = 0.384, p < 0.001), indicating partial mediation.

**Conclusion:**

Favourable perceptions of PIL-SMART were associated with higher teacher ratings of children’s science and environmental literacy. Because this cross-sectional study used teacher-reported measures, the findings indicate associations rather than causal effects. Future studies should use child assessments, classroom observations, and longitudinal or experimental designs.

## 1. Research background

The development of the 21 ^st^ century brings major changes in various aspects of life, including the field of education. An increasingly complex world, characterized by technological advances, environmental changes, and global challenges such as the climate crisis and the degradation of natural resources, demands the birth of a generation capable of thinking critically, adaptively, and responsibly ( Moffit, 2023; Şentürk, 2017). In this context, science literacy and environmental literacy are two fundamental competencies that need to be developed systematically from an early age ( Agal & Seal, 2025). The increasing complexity of contemporary social, technological, and environmental challenges has strengthened the need to develop science and environmental literacy from early childhood. Science literacy in early childhood involves more than remembering scientific facts; it includes children’s emerging abilities to observe, ask questions, investigate simple phenomena, interpret evidence, and communicate explanations based on concrete experiences ( Chaesar & Andayani, 2024). Environmental literacy similarly encompasses children’s developing knowledge, awareness, attitudes, and responsible behaviours toward their natural surroundings ( Isa et al., 2021). These two forms of literacy are closely connected because understanding basic scientific phenomena can help children recognize relationships among living things, natural resources, and human actions. Therefore, their development should begin through developmentally appropriate and contextually meaningful learning experiences.

Despite their importance, science and environmental learning in early childhood education continues to encounter pedagogical limitations. Classroom activities are frequently dominated by teacher-directed instruction in which children receive information without sufficient opportunities to investigate, experiment, or construct explanations. Learning domains such as science, mathematics, art, religion, language, and technology may also be presented separately, resulting in fragmented learning experiences that are not sufficiently connected to children’s everyday lives ( Amira & Kadir, 2025; Ramulumo, 2025). Consequently, children may have limited opportunities to relate scientific concepts to environmental issues or practise environmentally responsible behaviour through authentic activities. These conditions indicate the need for integrated learning that combines scientific inquiry, interdisciplinary content, and direct interaction with children’s surroundings.

Previous researchers have introduced several pedagogical solutions to address these problems. Inquiry-based learning has been used to engage children in questioning, observation, investigation, and evidence-based explanation, while project-based learning provides opportunities to explore meaningful problems over an extended period and produce tangible outcomes. Experiential and hands-on science activities have also been employed to connect abstract concepts with observable phenomena and increase children’s active participation ( Chaesar & Andayani, 2024; Lestari et al., 2025; Liberali et al., 2025). Meanwhile, integrated STEM or STEAM approaches have attempted to connect science and mathematics with technology, engineering, and art through interdisciplinary activities. Environmental and place-based learning has similarly emphasized direct interaction with plants, animals, water, soil, waste, and other elements of children’s immediate environments ( Irawan et al., 2024; Moffit, 2023).

Although these approaches have generated promising learning experiences, several limitations remain. Inquiry and project-based activities are frequently implemented as separate strategies rather than as components of a unified instructional framework. STEM and STEAM approaches may provide interdisciplinary learning but do not always incorporate moral, religious, or ecological values that influence early childhood education in the Indonesian context. Environmental education may also focus on knowledge or habituated behaviour without systematically connecting environmental understanding to scientific inquiry. Moreover, many previous studies have focused primarily on children’s achievement or classroom interventions, while teachers’ perceptions of the relevance, usability, and implementation of integrated learning models remain less frequently examined. This limitation is important because teachers ultimately interpret, adapt, and implement learning models in their classrooms.

Teachers’ perceptions can influence their willingness and consistency in adopting innovative pedagogical practices. When teachers perceive a model as relevant, understandable, feasible, and compatible with children’s developmental characteristics, they may be more likely to implement it consistently ( Chandra et al., 2023; Feng & Zhang, 2023; Williams et al., 2023). Conversely, perceived complexity, limited resources, or incompatibility with classroom conditions can constrain implementation. Examining teachers’ perceptions is therefore necessary for understanding the potential implementation of an integrated model. However, the literacy outcomes examined in the present study should be understood as teachers’ ratings of children’s science and environmental literacy rather than as direct assessments of children’s competencies.

To address these pedagogical and research gaps, the present study examines PIL-SMART, an integrated learning framework that combines Project–Inquiry Learning with Science, Mathematics, Art, Religion, and Technology. The framework is intended to connect project activities, inquiry processes, interdisciplinary content, values, and technology within learning experiences related to children’s everyday environments. In contrast to approaches that treat these components separately, PIL-SMART organizes them within an integrated framework through which children can observe phenomena, formulate questions, undertake simple investigations, communicate findings, create products, and reflect on scientific and environmental meanings. The model therefore offers a contextually relevant approach for connecting scientific exploration with environmental awareness in early childhood education.

Nevertheless, empirical evidence concerning PIL-SMART remains limited. In particular, few studies have simultaneously examined the relationships among teachers’ perceptions of PIL-SMART implementation, teachers’ ratings of children’s science literacy, and teachers’ ratings of children’s environmental literacy within one structural model. It also remains unclear whether perceived science literacy contributes to perceived environmental literacy and whether it serves as an indirect pathway between PIL-SMART and environmental literacy. Addressing this gap is urgent because an integrated model cannot be recommended solely on its conceptual promise; its measurement quality and relationships with relevant literacy constructs must be empirically examined.

Accordingly, this study investigates the associations among teachers’ perceptions of PIL-SMART implementation, teacher-rated science literacy, and teacher-rated environmental literacy in early childhood education. Structural Equation Modeling using the SEMLj module in Jamovi was employed to evaluate the measurement model and test the hypothesized structural relationships simultaneously. The study examines whether PIL-SMART is positively associated with science literacy and environmental literacy, whether science literacy is associated with environmental literacy, and whether science literacy provides an indirect pathway between PIL-SMART and environmental literacy. The study contributes by evaluating an interdisciplinary learning framework within the Indonesian early childhood education context while explicitly considering teachers’ perceptions as the source of measurement.

## 2. Literature review

### 2.1 PIL-SMART learning method

PIL-SMART refers to Project–Inquiry Learning integrating Science, Mathematics, Art, Religion, and Technology. In this abbreviation, “PIL” represents Project–Inquiry Learning, while “SMART” represents Science, Mathematics, Art, Religion, and Technology. The term should be written consistently as PIL-SMART throughout the article. In the present study, PIL-SMART is conceptualized as an interdisciplinary framework for organizing project and inquiry processes within early childhood learning activities that integrate scientific exploration, mathematical reasoning, artistic expression, religious or moral values, and age-appropriate uses of technology ( Lev et al., 2020; Siraj-Blatchford, 2016).

PIL-SMART draws on constructivist perspectives that regard children as active participants in developing understanding through interaction, exploration, and reflection. Within this framework, children are not positioned merely as recipients of information. They are encouraged to observe phenomena, formulate questions, make predictions, undertake simple investigations, discuss findings, produce representations or products, and reflect on the meaning of their experiences. Teachers facilitate these processes by organizing the learning environment, providing scaffolding, introducing relevant materials, and encouraging children to communicate their emerging explanations ( Odell & Kennedy, 2020; Strawhacker et al., 2020).

Project–Inquiry Learning constitutes the pedagogical foundation of PIL-SMART. Project learning allows children to engage with a meaningful topic or problem through interconnected activities over a particular period, whereas inquiry provides the investigative processes through which children formulate questions and develop evidence-based explanations. Combining the two approaches is intended to prevent projects from becoming activities focused only on producing products without sufficient investigation. It also prevents inquiry from becoming a series of isolated experiments that are disconnected from a broader learning purpose. Thus, project and inquiry processes operate as mutually reinforcing components of the model ( Nimmo & Park, 2009; Wight et al., 2016).

The SMART components provide the interdisciplinary content of the framework. Science involves observing, predicting, experimenting, and explaining natural phenomena. Mathematics includes classification, comparison, counting, measurement, pattern recognition, and spatial reasoning. Art allows children to express their understanding through drawing, movement, construction, music, or other symbolic forms. Religion refers to age-appropriate moral and spiritual values, including gratitude, responsibility, care, and respect for living things and the environment. Technology is used as a supporting tool for observation, documentation, information access, communication, or the creation of simple learning products rather than as a replacement for direct experience ( Daniele et al., 2025; Ramulumo, 2025).

The learning sequence of PIL-SMART consists of five interconnected stages. First, the teacher introduces a contextual phenomenon or problem that is familiar and meaningful to children. Second, children formulate questions and predictions through guided discussion. Third, children undertake project-based investigation by observing, comparing, experimenting, measuring, documenting, or collecting relevant information. Fourth, children integrate their findings through scientific explanation, mathematical representation, artistic expression, moral or religious reflection, and appropriate technological support. Fifth, children communicate their products or findings and reflect on the learning process with teachers and peers. These stages may be adapted according to children’s developmental characteristics, classroom conditions, available resources, and the topic being investigated.

For example, a project concerning plant growth may involve children observing seeds and plants, asking what plants need to grow, comparing plant height, documenting changes, drawing plant development, and discussing responsibility for caring for living things. Science is represented through observation and investigation, mathematics through measurement and comparison, art through visual representation, religion through gratitude and responsibility, and technology through photographic documentation or age-appropriate digital presentation. The example demonstrates that the SMART components are not separate subjects but complementary dimensions integrated within one project–inquiry learning experience.

In this study, teachers’ perceptions of PIL-SMART were operationalized through indicators concerning the model’s perceived relevance, clarity, feasibility, interdisciplinary integration, compatibility with children’s characteristics, support for active exploration, and potential contribution to classroom learning. Accordingly, the PIL-SMART construct represents teachers’ evaluations of the model’s implementation rather than a direct measure of implementation fidelity. This distinction is important because favourable perceptions do not necessarily demonstrate that all components of the model were implemented consistently in classroom practice.

### 2.2 Science literacy

Science literacy is understood as the ability of individuals to understand scientific concepts and processes and apply them in explaining phenomena, interpreting evidence, and making responsible decisions in daily life ( Aunillah, 2024; Rai et al., 2025). Science literacy is also interpreted as the active involvement of individuals in scientific issues based on scientific reasoning and evidence. The National Research Council emphasizes the importance of integrating conceptual knowledge and scientific practice, such as observing, questioning, investigating, and interpreting data ( Chandra et al., 2023; Hong et al., 2019). In addition, science literacy includes critical thinking and problem-solving skills in social and technological contexts ( Liana et al., 2024; Thiel, 2025).

In the context of early childhood education, science literacy can be developed from an early age through hands-on experience that is appropriate to the child’s developmental stage ( Fitriani, 2024). Science literacy is also seen as a means to shape social attitudes, values, and responsibilities, including concern for the environment ( Agal & Seal, 2025). Thus, science literacy is not only oriented towards mastering concepts, but also on developing process skills, scientific attitudes, and social awareness holistically.

H1: Teachers’ perceptions of the PIL-SMART learning model have a significant effect on science literacy in early childhood education.

### 2.3 Environmental literacy

Environmental literacy is understood as the ability of individuals to understand environmental systems and problems and show attitudes and behaviors that are responsible for preserving nature. Environmental literacy encompasses a combination of ecological knowledge, awareness, and skills in making decisions related to the environment ( Isa et al., 2021). In addition, environmental literacy emphasizes the integration of knowledge, thinking skills, caring attitudes, and pro-environmental behaviors as the core of its formation ( Masykuroh et al., 2024).

Environmental literacy is part of education for sustainable development that emphasizes the formation of value and responsibility towards nature from an early age ( Guerrero Fernández et al., 2022). Its development takes place effectively through direct experience and real interaction with the surrounding environment. In early childhood, environmental literacy is built through habituation, play activities, and strengthening attitudes that respect nature in daily life ( Feng & Zhang, 2023; J. Li et al., 2025). Therefore, environmental literacy focuses not only on conceptual understanding, but also on the formation of sustainable attitudes and behaviors as a whole.

H2: Teachers’ perceptions of the PIL-SMART learning model have a significant effect on environmental literacy in early childhood education.

H3: Science literacy has a significant effect on environmental literacy in early childhood education.

**Figure 1.**
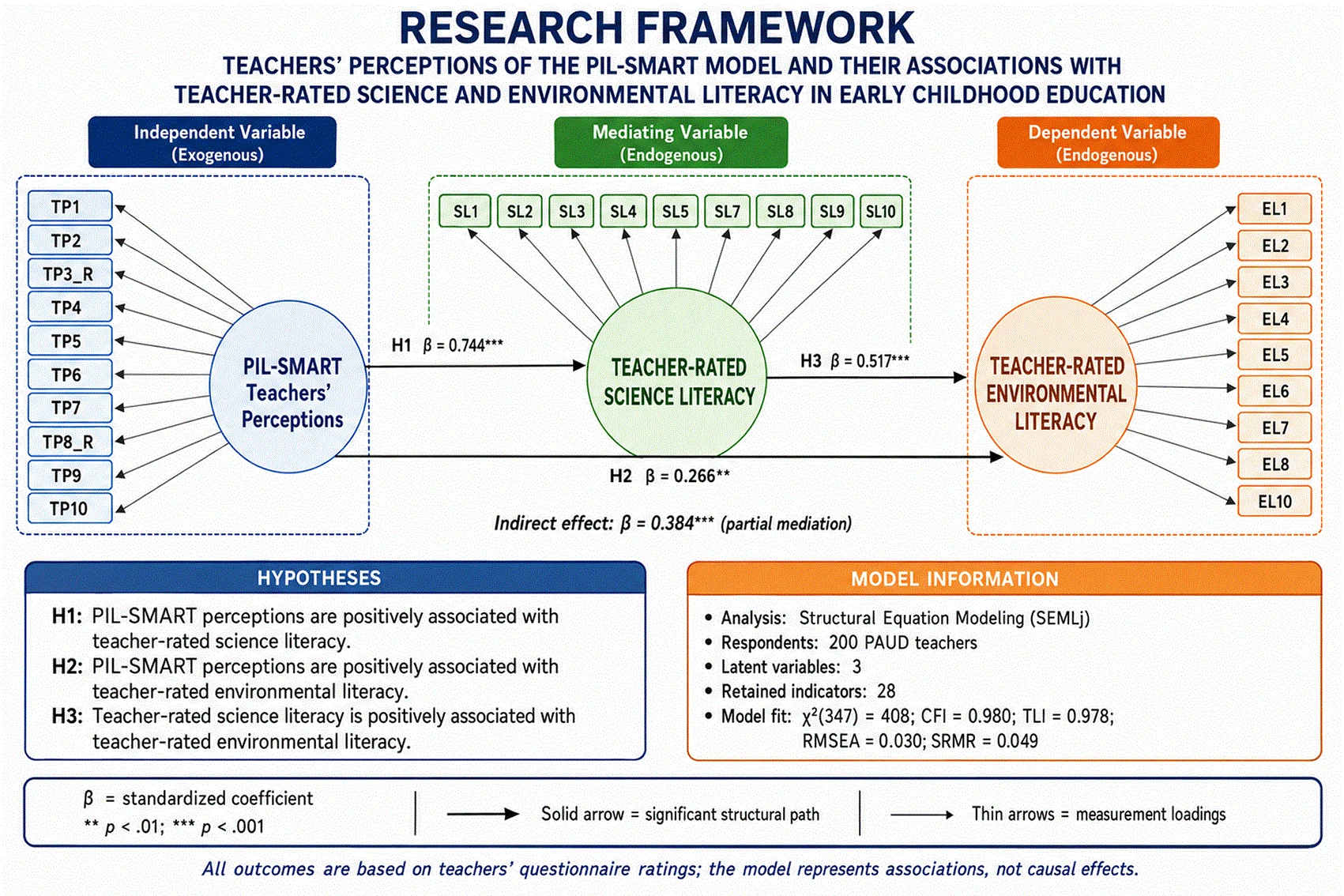
Research framework.

The conceptual framework of this study illustrates the structural relationships among the PIL-SMART learning model, science literacy, and environmental literacy in early childhood education, as presented in Figure 1. The framework explains that teachers’ perceptions of the implementation of the PIL-SMART model are assumed to influence the development of children’s science literacy and environmental literacy. In addition, science literacy is also hypothesized to contribute to environmental literacy development. The framework was developed based on theories of inquiry learning, project-based learning, and literacy development in early childhood education. Therefore, Figure 1 serves as the basis for testing the relationships among variables through the Structural Equation Modeling (SEM) analysis used in this study.

## 3. Research methods

### 3.1 Samples and procedures

This study employed a quantitative cross-sectional survey design to examine the associations among teachers’ perceptions of PIL-SMART, teacher-rated science literacy, and teacher-rated environmental literacy in early childhood education. A cross-sectional design was selected because all variables were measured at one point in time using structured questionnaire responses. Accordingly, the structural relationships identified in this study were interpreted as statistical associations rather than causal effects.

The study was conducted in Bekasi City, West Java Province, Indonesia. Participants were recruited from 125 early childhood education institutions located in the city. Purposive sampling was employed to select respondents who met the study criteria. Eligible respondents were teachers who were actively teaching in an early childhood education institution and had experience implementing science- and environment-related learning activities. Participation was voluntary, and teachers who did not meet these criteria or did not provide informed consent were not included.

A total of 200 early childhood education teachers participated in the study. The sample size was reported transparently in relation to the complexity of the final Structural Equation Modeling model, which contained three latent constructs, 28 retained indicators, and 87 freely estimated parameters. Rather than relying solely on a universal respondent-to-parameter rule, model estimation was evaluated by examining convergence, standardized factor loadings, improper solutions, residual variances, and multiple model-fit indices. Nevertheless, the sample size and non-probability sampling method were recognized as limitations when interpreting the stability and generalizability of the results.

Data were collected using a structured questionnaire administered directly or through an online survey platform. The questionnaire measured three constructs: teachers’ perceptions of PIL-SMART, teachers’ ratings of children’s science literacy, and teachers’ ratings of children’s environmental literacy. Therefore, science and environmental literacy were not assessed through direct tests or systematic observations of children. Before completing the questionnaire, participants received information concerning the study objectives, participation procedures, voluntary nature of participation, confidentiality protections, and their right to withdraw without penalty.

The research procedure consisted of four stages. First, questionnaire indicators were developed based on a review of literature concerning integrated learning models, science literacy, and environmental literacy in early childhood education. Second, the content validity and clarity of the indicators were examined through consultation with experts in early childhood education and educational evaluation. Third, the questionnaire was distributed to eligible teachers from participating early childhood education institutions. Fourth, the collected data were screened, coded, and analysed using reliability analysis, Confirmatory Factor Analysis, and Structural Equation Modeling. Negatively worded items were reverse-coded before analysis so that higher scores consistently represented higher levels of the respective constructs.

Following the measurement-model evaluation, SL6 was excluded because it demonstrated a standardized factor loading of 0.330 and did not adequately represent the science literacy construct. EL9_R was excluded because its inclusion resulted in a non-positive-definite sample covariance matrix and prevented stable model estimation. The final model therefore retained 28 indicators: ten indicators for PIL-SMART, nine indicators for science literacy, and nine indicators for environmental literacy. All item-retention and removal decisions were based on a combination of statistical evidence, model-estimation diagnostics, and theoretical considerations.

Prior to data collection, ethical approval was obtained from the Ethical Clearance Commission of the Research and Community Service Institute (LPPM), Universitas Panca Sakti Bekasi, Indonesia, under Ethical Clearance Certificate No. 001/LPPM.ECC/PSUB/V/2026. All participants were informed about the study objectives, voluntary participation, anonymity, confidentiality, and their right to decline or discontinue participation. Verbal informed consent was obtained before questionnaire administration, and the verbal consent procedure was included in the protocol approved by the institutional ethics committee. This procedure was used because the study involved minimal-risk questionnaire participation, did not collect sensitive personal data, and did not involve intervention or direct assessment of children. Participant agreement was documented by the data-collection team before access to the questionnaire was provided.

### 3.2 Respondent profile

The study involved 200 early childhood education teachers from 125 PAUD institutions in Bekasi City, West Java Province, Indonesia. Respondent characteristics were examined in terms of gender, age, educational attainment, and teaching experience. These characteristics provide contextual information for interpreting teachers’ perceptions of PIL-SMART and their ratings of children’s science and environmental literacy.

Most respondents were women, reflecting the predominantly female composition of the teaching workforce in the participating early childhood education institutions. In terms of age, most respondents were between 25 and 40 years old. However, gender and age were treated only as demographic characteristics and were not interpreted as indicators of competence or the quality of PIL-SMART implementation.

Regarding educational attainment, most respondents held a bachelor’s degree in early childhood education or another relevant educational field. Some respondents had also participated in professional-development activities related to innovative, science-based, environmental, or contextual learning. These educational and professional experiences provided relevant background for responding to questionnaire items concerning integrated early childhood learning.

Most respondents had between five and ten years of teaching experience, while the remaining participants had either shorter or longer teaching experience. This distribution indicates that the study involved teachers with varying levels of professional experience. Nevertheless, teaching experience alone does not demonstrate that respondents had implemented all components of PIL-SMART consistently. The data therefore represent teachers’ perceptions of the framework and their ratings of children’s literacy-related abilities rather than direct evidence of implementation fidelity.

### 3.3 Measurement analysis using SEMLj

The data analysis in this study was carried out using Structural Equation Modeling (SEM) with the help of Jamovi software through the SEMLj module. The SEM method is used because it is able to analyze the complex relationships between several latent variables simultaneously. In addition, SEM also allows researchers to test measurement models and structural models simultaneously. This analysis was used to determine the relationship between teachers’ perception of the Pil-Smart learning model and science literacy and environmental literacy in early childhood.

The research model consists of three latent constructs, namely Pil-Smart, science literacy, and environmental literacy. The Pil-Smart construct is measured using ten indicators that describe teachers’ perceptions of the implementation of the learning model. The construct of science literacy is also measured using ten indicators that describe children’s ability to understand simple science concepts. Meanwhile, the construct of environmental literacy is measured using ten indicators related to children’s awareness and behavior towards the surrounding environment. All indicators are measured using a five-point Likert scale.

The SEM analysis stage is carried out through several steps. The first step is to test the measurement model to determine the validity of the construct through the analysis of the loading factor on each indicator. The second step is the evaluation of the feasibility of the model using various goodness of fit indices such as Chi-square, RMSEA, SRMR, CFI, and TLI. The third step is to test the structural model to find out the relationships between latent variables using the values of the path coefficient and the level of significance.

In addition, the analysis also includes testing the coefficient of determination (R ^2^) to find out the extent to which the dependent variables can be explained by the research model. The results of the SEM analysis were then interpreted to explain the relationship between the Pil-Smart learning model and science literacy and environmental literacy in early childhood. The entire analysis process is carried out using the latest version of Jamovi software equipped with the SEMLj module. Thus, the analysis method used is able to provide comprehensive results in explaining the relationship between research variables.

## 4. Research results

### 4.1 Model fit evaluation

The final Structural Equation Model was estimated using Maximum Likelihood estimation with the NLMINB optimization method. The model included 200 observations, three latent constructs, 28 retained indicators, and 87 freely estimated parameters. The estimation process converged successfully after 42 iterations and produced no negative latent variances or other improper solutions. The final model was overidentified, with 347 degrees of freedom, and was therefore not a saturated model.

The chi-square test for the final model was statistically significant, χ ^2^(347) = 408, p = 0.014. However, the chi-square statistic is sensitive to sample size and model complexity and should therefore be interpreted together with other model-fit indices. The relative chi-square value was 1.18, which was below the commonly applied threshold of 3.00 and indicated an acceptable correspondence between the hypothesized model and the observed covariance matrix.

The incremental and residual-based fit indices provided additional support for the model. The Comparative Fit Index was 0.980, and the Tucker–Lewis Index was 0.978. Both values exceeded the recommended threshold of 0.90 and the more stringent threshold of 0.95. The Root Mean Square Error of Approximation was 0.030, with a 95% confidence interval ranging from 0.014 to 0.041, while the Standardized Root Mean Square Residual was 0.049. These values were below the recommended maximum of 0.08 and indicated a low level of approximation and standardized residual error.

Some secondary incremental indices were lower than 0.90, including the Normed Fit Index of 0.880 and the Relative Fit Index of 0.869. Nevertheless, the principal fit indices—CFI, TLI, RMSEA, and SRMR—consistently supported the adequacy of the final model. Accordingly, the model was considered sufficiently fitted for interpreting the measurement and structural relationships. The results were not described as a “perfect fit,” because model evaluation was based on the combined evidence from multiple indices rather than a single fit statistic.

To examine whether all indicators could be explained by one general factor, the hypothesized model was compared with an alternative single-factor model. The single-factor model demonstrated poor fit, χ ^2^(350) = 940, p < 0.001, CFI = 0.805, TLI = 0.789, RMSEA = 0.092, and SRMR = 0.081. In comparison, the final three-construct model produced substantially better fit, supporting the empirical distinction among teachers’ perceptions of PIL-SMART, teacher-rated science literacy, and teacher-rated environmental literacy.

Table 1 presents the results of the model fit evaluation obtained from the Structural Equation Modeling (SEM) analysis.

**Table 1.** Model fit evaluation.

Model	χ ^2^	df	χ ^2^/df	CFI	TLI	RMSEA (95% CI)	SRMR
Final three-construct model	408	347	1.18	0.980	0.978	0.030 (0.014–0.041)	0.049
Alternative single-factor model	940	350	2.69	0.805	0.789	0.092 (0.085–0.099)	

The RMSEA value presented in Table 2 was 0.030, with a 95% confidence interval ranging from 0.014 to 0.041. This value was below the recommended threshold of 0.08, indicating a low level of approximation error. The SRMR value was 0.049, which also satisfied the acceptable criterion of less than 0.08. As shown in Table 3, the Comparative Fit Index (CFI) was 0.980 and the Tucker–Lewis Index (TLI) was 0.978. Both values exceeded the recommended threshold of 0.90 and the more stringent criterion of 0.95, providing strong support for the fit of the final model. Although the NFI (0.880) and RFI (0.869) were marginally below 0.90, the combined evidence from the relative chi-square (χ ^2^/df = 1.18), CFI, TLI, RMSEA, and SRMR indicated that the final three-construct model adequately represented the observed data and was appropriate for interpreting the structural relationships.

**Table 2.** Goodness-of-fit indices for the final structural model.

Fit index	Final model	95% CI	Recommended criterion	Interpretation
χ ^2^/df	1.18	—	≤3.00	Good fit
SRMR	0.049	—	≤0.08	Good fit
RMSEA	0.030	0.014–0.041	≤0.08	Good fit
CFI	0.980	—	≥0.90	Good fit
TLI	0.978	—	≥0.90	Good fit
NFI	0.880	—	≥0.90	Marginal
RFI	0.869	—	≥0.90	Marginal
IFI	0.980	—	≥0.90	Good fit
PNFI	0.808	—	Higher values indicate greater parsimony	Acceptable

**Table 3.** Comparative model fit indices.

Model	χ ^2^	df	χ ^2^/df	CFI	TLI	NFI	RFI	IFI	RMSEA (95% CI)	SRMR
Final three-construct model	408	347	1.18	0.980	0.978	0.880	0.869	0.980	0.030 (0.014–0.041)	0.049
Alternative single-factor model	940	350	2.69	0.805	0.789	0.723	0.701	0.807	0.092 (0.085–0.099)	0.081
Difference (Δ)	−532	−3	−1.51	+0.175	+0.189	+0.157				

Table 2 and Table 3 present the goodness-of-fit indices used to evaluate the suitability of the SEM model.

The RMSEA value shown in Table 2 was 0.002, which was below the recommended threshold of 0.08, indicating a very small approximation error. Furthermore, the SRMR value was 0.074, which also satisfied the acceptable criterion of less than 0.08. As shown in Table 3, the Comparative Fit Index (CFI) value was 0.991 and the Tucker-Lewis Index (TLI) value was 0.990. Both indices exceeded the recommended value of 0.90, indicating that the model had an excellent fit with the observed data. Overall, these findings confirmed that the proposed SEM model was feasible and suitable for structural analysis.

### 4.2 Measurement model

Table 4 presents the factor loading results for the measurement model used in this study.

**Table 4.** Measurement model results.

Latent construct		Indicator	Unstandardized estimate	SE	95% CI	Standardized loading (β)	z	p
PIL-SMART		TP1	1.000	—	—	0.739	—	—
		TP2	0.995	0.093	0.813–1.180	0.747	10.68	<0.001
		TP3_R	1.099	0.105	0.894–1.300	0.734	10.48	<0.001
		TP4	1.053	0.100	0.857–1.250	0.738	10.56	<0.001
		TP5	1.120	0.099	0.926–1.310	0.790	11.36	<0.001
		TP6	1.048	0.095	0.862–1.230	0.770	11.04	<0.001
		TP7	1.090	0.096	0.901–1.280	0.789	11.34	<0.001
		TP8_R	1.058	0.096	0.870–1.250	0.770	11.05	<0.001
		TP9	1.148	0.102	0.948–1.350	0.783	11.24	<0.001
		TP10	1.036	0.100	0.840–1.230	0.725	10.35	<0.001
Science literacy		SL1	1.000	—	—	0.717	—	—
		SL2	0.988	0.100	0.792–1.180	0.730	9.91	<0.001
		SL3	0.892	0.097	0.703–1.080	0.681	9.24	<0.001
		SL4	1.021	0.103	0.819–1.220	0.732	9.93	<0.001
		SL5	0.951	0.097	0.761–1.140	0.725	9.83	<0.001
		SL7	0.895	0.102	0.695–1.100	0.646	8.76	<0.001
		SL8	0.983	0.103	0.781–1.180	0.705	9.57	<0.001
		SL9	0.812	0.100	0.615–1.010	0.596	8.08	<0.001
		SL10	0.968	0.096	0.781–1.160	0.745	10.11	<0.001
Environmental literacy		EL1	1.000	—	—	0.719	—	—
		EL2	0.997	0.107	0.787–1.210	0.687	9.31	<0.001
		EL3	1.025	0.110	0.809–1.240	0.685	9.29	<0.001
		EL4	1.003	0.106	0.796–1.210	0.700	9.49	<0.001
		EL5	1.052	0.110	0.837–1.270	0.706	9.58	<0.001
		EL6	1.061	0.107	0.851–1.270	0.728	9.88	<0.001
		EL7	1.036	0.108	0.824–1.250	0.705	9.57	<0.001
		EL8	1.087	0.113	0.866–1.310	0.708	9.60	<0.001
		EL10	1.107	0.110	0.891–1.320	0.741	10.05	<0.001	

The measurement-model results showed that all 28 retained indicators adequately represented their respective latent constructs, with standardized factor loadings ranging from 0.596 to 0.790. Within the PIL-SMART construct, TP5 demonstrated the highest standardized loading (β = 0.790), followed by TP7 (β = 0.789) and TP9 (β = 0.783), while all ten indicators had loadings above 0.70. For teacher-rated science literacy, the standardized loadings ranged from 0.596 to 0.745, with SL10 providing the strongest contribution (β = 0.745) and SL9 showing the lowest but still acceptable loading (β = 0.596). In the teacher-rated environmental literacy construct, EL10 demonstrated the highest loading (β = 0.741), whereas EL3 had the lowest loading (β = 0.685), and all retained indicators were statistically significant at p < 0.001. SL6 was excluded because its initial standardized loading was only 0.330, while EL9_R was excluded because its inclusion resulted in a non-positive-definite sample covariance matrix. Overall, the magnitude and statistical significance of the retained factor loadings supported the adequacy of the final measurement model for representing teachers’ perceptions of PIL-SMART and their ratings of children’s science and environmental literacy.

### 4.3 Structural model

**Figure 2.**
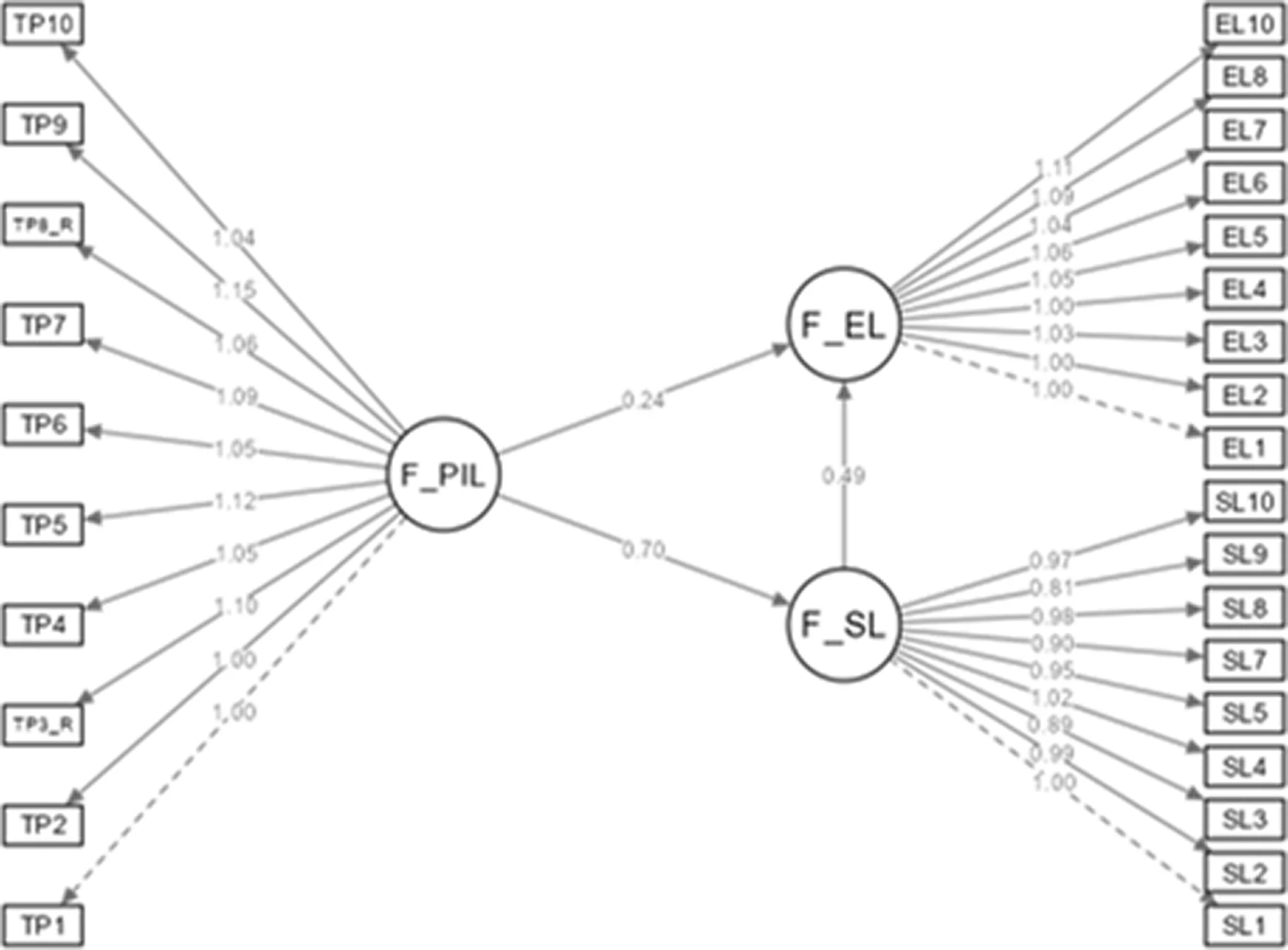
Structural model of pil-smart, science literacy, and environmental literacy.

Figure 2 illustrates the structural relationships among Pil-Smart, science literacy, and environmental literacy in the SEM model.

Figure 2 illustrates the final measurement and structural model connecting teachers’ perceptions of PIL-SMART, teacher-rated science literacy, and teacher-rated environmental literacy. As shown in Figure 2, PIL-SMART was positively associated with science literacy (unstandardized estimate = 0.701; standardized β = 0.744; p < 0.001) and environmental literacy (unstandardized estimate = 0.238; standardized β = 0.266; p = 0.006). Science literacy was also positively associated with environmental literacy (unstandardized estimate = 0.489; standardized β = 0.517; p < 0.001). Therefore, H1, H2, and H3 were supported. The indirect association between PIL-SMART and environmental literacy through science literacy was statistically significant (unstandardized estimate = 0.343; standardized β = 0.384; p < 0.001), indicating partial mediation. The model retained 28 indicators, comprising ten PIL-SMART indicators, nine science-literacy indicators, and nine environmental-literacy indicators. Overall, Figure 2 provides visual support for the positive direct and indirect associations identified in the final structural model.

The structural-model results showed that teachers’ perceptions of PIL-SMART were positively and significantly associated with teacher-rated science literacy (β = 0.744, p < 0.001). This result indicates that more favourable teacher perceptions of PIL-SMART were associated with higher teacher ratings of children’s science literacy; therefore, H1 was supported. Teachers’ perceptions of PIL-SMART were also positively and significantly associated with teacher-rated environmental literacy (β = 0.266, p = 0.006), supporting H2. Although this direct relationship was weaker than the association between PIL-SMART and science literacy, it indicates that PIL-SMART perceptions retained a statistically significant relationship with environmental literacy after science literacy was included in the model.

Teacher-rated science literacy was positively and significantly associated with teacher-rated environmental literacy (β = 0.517, p < 0.001); therefore, H3 was supported. The indirect association between PIL-SMART and environmental literacy through science literacy was also statistically significant (β = 0.384, p < 0.001), while the total association was β = 0.651 (p < 0.001). Because both the direct and indirect paths were significant, teacher-rated science literacy demonstrated a partial mediating role in the association between teachers’ perceptions of PIL-SMART and teacher-rated environmental literacy. Overall, the final structural model presented in Figure 2 supported all three hypotheses and demonstrated positive relationships among the three latent constructs. However, these findings represent associations based on teachers’ questionnaire ratings and should not be interpreted as evidence of causal effects or direct improvements in children’s literacy.

## 5. Discussion

The results of this study show that the Pil-Smart learning model has a significant influence on science literacy in early childhood education. These findings show that an exploratory and hands-on experience learning approach is able to improve children’s understanding of basic science concepts. In early childhood education, the learning process involving concrete activities is very important because children learn through interaction with the surrounding environment. The Pil-Smart model provides children with the opportunity to observe and explore directly. Through these activities, children can develop curiosity and scientific thinking skills from an early age. Therefore, this learning model can be an effective strategy in supporting the development of science literacy in children.

The findings of this study are in line with various previous studies that show that experiential learning can improve children’s science literacy skills. Early childhood tends to understand science concepts through exploratory activities such as observing, trying, and experimenting ( Cutrer-Párraga et al., 2020; Moffit, 2023; Quro & Choiriyah, 2022). These activities help children understand the causal relationships that occur in natural phenomena. Learning that emphasizes hands-on experience can also increase children’s involvement in the learning process ( Brandt et al., 2025; Smolkin & Donovan, 2015). In addition, exploration activities allow children to develop critical thinking skills from an early age. Thus, an exploratory learning approach has great potential in improving science literacy ( Singh, 2022; Thiel, 2025).

The results of this study also show that teachers’ perceptions of learning models have an important role in the early childhood learning process. Teachers who have a positive perception of innovative learning models tend to be more active in implementing creative learning strategies ( Aunillah, 2024; Şentürk, 2017). Teachers’ perceptions can influence how they design learning activities that actively engage children. In early childhood education, teachers play the role of facilitators who help children find knowledge through learning experiences ( Hong et al., 2019). Therefore, teachers’ understanding of the learning model is an important factor in the successful implementation of the model. These findings show that teacher competency development is indispensable in the implementation of innovative learning models.

However, the results of this study show that the Pil-Smart model does not have a significant direct influence on environmental literacy. These findings suggest that the development of environmental literacy in early childhood is likely influenced by a variety of other factors outside of the learning model used ( Clark et al., 2020; Salleh & Omar, 2025). Environmental literacy is not only related to knowledge, but also related to attitudes and behaviors towards the environment. Forming an attitude of caring for the environment usually requires a longer and more continuous learning process. In addition, children’s experiences in daily life can also affect the development of their environmental literacy ( Suharti et al., 2020). Therefore, environmental learning needs to be carried out consistently in various learning activities.

The relationship between science literacy and environmental literacy found in this study shows that there is a relationship between the two concepts. Understanding science concepts can help children understand environmental phenomena that occur around them ( An et al., 2019; Rafiq-uz-Zaman, 2025). For example, an understanding of water, soil, and plants can help children understand the importance of taking care of the environment. This knowledge can help children understand the relationship between humans and nature. By understanding the basic concepts of science, children can develop awareness of the importance of protecting the environment. Therefore, science literacy can be an important basis for the development of environmental literacy in early childhood.

These findings suggest that the integration of science and environmental learning is essential in early childhood education. Learning that combines science concepts with activities related to the environment can help children understand the relationship between humans and nature more comprehensively. Activities such as planting plants, observing animals, or keeping the environment clean can help children understand these concepts concretely. Contextual learning activities can also increase children’s involvement in the learning process. In addition, these activities can also help children develop an attitude of caring for the environment from an early age ( Britsch, 2017; Robertson & Moran, 2019). Therefore, the integration of science and environmental learning needs to be strengthened in learning activities.

The Pil-Smart model provides opportunities for teachers to develop more creative and contextual learning activities. The learning approach used in this model encourages children to be active in the learning process. Children not only receive information passively but also engage in exploratory activities. These activities help children build knowledge through learning experiences ( Daniele et al., 2025). In addition, an interactive learning approach can also increase children’s motivation to learn. Thus, the Pil-Smart model can be an effective learning strategy in early childhood education.

The findings of this study also provide practical implications for early childhood education teachers. Teachers need to design learning activities that are able to integrate various aspects of learning holistically. Learning that combines science concepts with activities related to the environment can help children understand natural phenomena better. In addition, learning activities also need to be designed to suit the characteristics of early childhood development ( Chen & Delaney, 2025; L. Li & Tan, 2015). Teachers can use a variety of activities such as simple experiments and environmental observation activities. Thus, learning can be more interesting and meaningful for children.

In the context of early childhood education, literacy development is one of the important aspects of the learning process. Literacy is not only related to the ability to read and write but also includes the ability to understand the world around children ( Daniele et al., 2025; Ramulumo, 2025). Science literacy and environmental literacy are an important part of developing these skills. Literacy development from an early age can help children develop critical thinking skills ( Ly-Hoang, 2023a, 2023b). In addition, literacy also helps children understand the relationship between humans and the environment. Therefore, early childhood education needs to pay greater attention to literacy development.

This research also contributes to the development of academic studies on innovative learning models in early childhood education. This study shows that a systematically designed learning model can have an impact on children’s literacy development. In addition, this study also shows that teacher perception is an important factor in the successful implementation of the learning model. These findings reinforce the importance of teacher competency development in implementing innovative learning approaches ( Marufah et al., 2025; Tariq, 2025). Thus, this research contributes to enriching the study of learning innovations in early childhood education.

Although this research makes an important contribution, there are some limitations that need to be considered. This study only involved a number of respondents from certain early childhood education institutions. Therefore, the results of the study may not be able to be generalized widely. In addition, this study uses a survey approach that relies on respondents’ perceptions ( Ly-Hoang, 2023b; Preston, 2018). Teachers’ perceptions can be influenced by their personal experiences as well as the context of their work environment. Therefore, further research can involve a wider sample to obtain more representative results.

Further research can also develop a more integrated learning model between science and the environment. Such integration can help children understand the relationship between humans and nature more comprehensively ( Daniele et al., 2025; Ly-Hoang, 2023a). In addition, further research can also use a mixed method approach to gain a deeper understanding of the implementation of learning models. The approach allows researchers to combine quantitative and qualitative data ( Abraham, 2021; Ly-Hoang, 2023b). Thus, the results of the research can provide a more comprehensive picture of the learning process. Follow-up research can also examine the influence of other factors that affect children’s environmental literacy.

Overall, this study shows that the Pil-Smart learning model has the potential to improve science literacy in early childhood education. Although its influence on environmental literacy is not significant, the relationship between science literacy and environmental literacy shows a connection between the two concepts. This shows that science learning can be the basis for the development of environmental awareness in children. Therefore, the integration of science and environmental learning needs to continue to be developed in early childhood education. The development of innovative learning models can help create a more meaningful learning experience for children. Thus, this research makes an important contribution to the development of learning strategies in early childhood education.

## 6. Conclusion

This study examined the associations among teachers’ perceptions of PIL-SMART, teacher-rated science literacy, and teacher-rated environmental literacy in early childhood education. The final Structural Equation Model demonstrated adequate fit with the observed data, χ ^2^(347) = 408, CFI = 0.980, TLI = 0.978, RMSEA = 0.030, and SRMR = 0.049. The measurement results also supported the reliability and overall construct quality of the 28 retained indicators representing the three latent constructs.

Teachers’ perceptions of PIL-SMART were positively and significantly associated with teacher-rated science literacy (β = 0.744, p < 0.001) and teacher-rated environmental literacy (β = 0.266, p = 0.006). Teacher-rated science literacy was also positively and significantly associated with teacher-rated environmental literacy (β = 0.517, p < 0.001). Accordingly, H1, H2, and H3 were supported. Science literacy also partially mediated the association between PIL-SMART and environmental literacy, as demonstrated by the significant indirect association (β = 0.384, p < 0.001). These findings suggest that teachers who evaluated PIL-SMART more favourably also tended to provide higher ratings of children’s science and environmental literacy.

The findings highlight the potential relevance of integrating project–inquiry learning with science, mathematics, art, religion, and technology in early childhood education. However, the results should not be interpreted as evidence that PIL-SMART directly caused improvements in children’s literacy because the study employed a cross-sectional survey and measured literacy through teachers’ questionnaire ratings rather than direct child assessments. Future research should evaluate PIL-SMART through experimental or longitudinal designs, direct assessments and classroom observations, larger and more geographically diverse samples, and systematic measures of implementation fidelity. Such studies are needed before firm conclusions can be drawn regarding the effectiveness and broader applicability of PIL-SMART in early childhood education.

## Ethical considerations

Ethical approval for this study was obtained from the Ethical Clearance Commission, Research Institutions and Community Service (LPPM), Panca Sakti University Bekasi, Indonesia, under Ethical Clearance Certificate No. 001/LPPM.ECC/PSUB/V/2026. The study complied with institutional ethical standards for research involving human participants. Informed consent was obtained from all participants prior to data collection.

## Data Availability

Zenodo: Analysis Result Data for Research Entitled Teachers’ Perceptions of The PIL-SMART Model: A SEMLJ Analysis Of Its Influence on Science and Environmental Literacy in Early Childhood Education https://doi.org/10.5281/zenodo.20305973 ( Priyanti et al., 2026). This project contains the following extended data:
1.
data_kuesioner_SEM_PLS_200_responden.xlsx 2. Structural Equation Modelling.omv 3. Structural Equation Modelling.pdf
