# Supplementary material for: Teachers' Perceptions of the PIL-SMART Model: A Semlj Analysis of its Influence on Science and Environmental Literacy in Early Childhood Education
Source: F1000Res. 2026 Aug 19;15:1034. Originally published 2026 Jun 30. [Version 2] doi: 10.12688/f1000research.181997.2 (PMC13560012; doi:10.12688/f1000research.181997.2)
Supplement: Peer review history

# Reviewer report: Reviewer response for version 2

**DOI:** 10.5256/f1000research.207997.r513435

**Published:** 2026-09-08

**Authors:** Dwi Ismawati

**Affiliations:** 1 University of Bengkulu, Bengkulu, Indonesia

 Project-Inquiry Learning with Science, Mathematics, Art, Religion and Technology is interesting. The strengths of the study are ethical approval, use of SEM and availability of underlying data. The manuscript will require substantial revision to be considered scientifically sound. There is inconsistency between Results and Discussion especially when considering the significance of the relationship between PIL-SMART and environmental literacy. The manuscript also reports contradictory model-fit statistics, requires clearer justification for item removal and estimator selection, and should provide more complete evidence of reliability, convergent validity, and discriminant validity. In addition, causal language should be avoided because the study uses a cross-sectional design and teacher-reported measures. The authors should also clarify whether PIL-SMART was actually implemented or only evaluated through teachers’ perceptions, address potential common-method bias and clustering effects, and strengthen the theoretical novelty of the model. Overall, the manuscript has potential, but I recommend Major Revision before it can be considered for publication.

Is the work clearly and accurately presented and does it cite the current literature?

Partly

If applicable, is the statistical analysis and its interpretation appropriate?

Partly

Are all the source data underlying the results available to ensure full reproducibility?

Yes

Is the study design appropriate and is the work technically sound?

Partly

Are the conclusions drawn adequately supported by the results?

Partly

Are sufficient details of methods and analysis provided to allow replication by others?

Partly

Reviewer Expertise:

Nonformal Education, Educational Technology, Learning Innovation, Science and Environmental Literacy, and Educational Research Methodology.

I confirm that I have read this submission and believe that I have an appropriate level of expertise to confirm that it is of an acceptable scientific standard, however I have significant reservations, as outlined above.

# Reviewer report: Reviewer response for version 2

**DOI:** 10.5256/f1000research.207997.r513434

**Published:** 2026-08-26

**Authors:** Abdulkadir rahardjanto

**Affiliations:** 1 University of Muhammadiyah Malang, Malang, East Java, Indonesia

This revised version incorporates the suggestions and feedback provided. The authors have carefully considered this input and used it to improve the quality of their manuscript. In my opinion, the manuscript is now suitable for acceptance. I also note that they have considered and adopted the feedback from the other reviewer—which was also crucial—as a basis for the revisions.

Is the work clearly and accurately presented and does it cite the current literature?

Yes

If applicable, is the statistical analysis and its interpretation appropriate?

Yes

Are all the source data underlying the results available to ensure full reproducibility?

Yes

Is the study design appropriate and is the work technically sound?

Yes

Are the conclusions drawn adequately supported by the results?

Yes

Are sufficient details of methods and analysis provided to allow replication by others?

Yes

Reviewer Expertise:

Biology Education, Biology, Ecology, Evironmental education and literacy

I confirm that I have read this submission and believe that I have an appropriate level of expertise to confirm that it is of an acceptable scientific standard.

# Reviewer report: Reviewer response for version 2

**DOI:** 10.5256/f1000research.207997.r512553

**Published:** 2026-08-25

**Authors:** Husamah H

**Affiliations:** 1 University of Muhammadiyah, Malang East, Java, Indonesia

I have carefully reviewed the revised manuscript and noted that the authors have responded thoroughly and constructively to my previous comments, as well as to those raised by the other reviewer. The revisions have been incorporated appropriately and have substantially improved the manuscript’s clarity, methodological transparency, coherence, and overall scholarly quality. The authors have also provided adequate explanations and supporting information for the issues previously identified. Therefore, I am satisfied with the revised version and recommend changing the peer-review status from **“Approved with Reservations”** to **“Approved.”**

Is the work clearly and accurately presented and does it cite the current literature?

Partly

If applicable, is the statistical analysis and its interpretation appropriate?

Yes

Are all the source data underlying the results available to ensure full reproducibility?

Yes

Is the study design appropriate and is the work technically sound?

Yes

Are the conclusions drawn adequately supported by the results?

Yes

Are sufficient details of methods and analysis provided to allow replication by others?

Yes

Reviewer Expertise:

Biology Education, Environmental Literacy, Ecology, And Sustainability Science

I confirm that I have read this submission and believe that I have an appropriate level of expertise to confirm that it is of an acceptable scientific standard.

# Reviewer report: Reviewer response for version 1

**DOI:** 10.5256/f1000research.200894.r501322

**Published:** 2026-08-03

**Authors:** Moleboheng Mokhele

**Affiliations:** 1 North - West University, Potchefstroom, South Africa

I have to say, this excited me when I started reading it, teacher perceptions of an integrated learning model, and how that connects to literacy outcomes, is genuinely something that hasn't been studied enough. It's the kind of gap I love to see someone finally taking on. And using SEM to test several relationships at once was the right call for a question this layered.

 But once I got into the details, I found a number of problems, some statistical, some conceptual, some just about how things are presented, that need to be fixed before this is ready to be published. My recommendation is major revisions, not because the idea isn't good, it's a great idea, but because the execution needs work.

**1. The direction of the results doesn't match what the paper says, and this is the biggest problem**

 The results show:

- PIL-SMART → Science literacy: β = −0.125, p = 0.043
- PIL-SMART → Environmental literacy: β = 0.0469, p = 0.091 (not significant)
- Science literacy → Environmental literacy: β = −0.1004, p = 0.010

Both of the significant results are negative. But the Abstract, Results, Discussion, and Conclusion all talk about these relationships as if they're positive and beneficial, saying things like "PIL-SMART is effective in improving science literacy" and "children who showed better science literacy also showed stronger environmental literacy."

 A negative number for PIL-SMART → science literacy actually means the opposite: higher teacher perception scores go with *lower* science literacy scores. That's not what the paper argues throughout the Discussion. There are three possibilities here: either this is a typing/output mistake and the sign should be positive, or the relationship really is negative and the whole interpretation needs to be rewritten, or the items were reverse-coded and this was never explained to the reader. This needs to be sorted out and explained clearly before anything else, right now the numbers and the story being told about them directly contradict each other, and any reviewer who understands statistics will catch this immediately.

**2. The model fit looks too perfect**

 χ² = 402 with df = 402 (so χ²/df = 1.00 exactly) and RMSEA = 0.002 is an unusually good fit, almost too good to be real. In practice, numbers this clean in real survey data are rare, and they usually point to one of a few problems: the model may be under-identified or saturated, the degrees of freedom may have been calculated wrong, or there were estimation issues that got hidden instead of fixed. The paper should show exactly how the degrees of freedom were calculated, confirm the model isn't saturated, and ideally include other fit measures alongside a sensitivity check. As it stands, this number will make reviewers suspicious rather than reassured.

**3. Important quality checks for the measurements are missing**

 For a paper using SEM, readers would normally expect to see:

- Cronbach's alpha or composite reliability for each construct
- Average variance extracted (AVE) to show convergent validity
- Discriminant validity checks (like the Fornell-Larcker criterion or HTMT ratio)

Only individual item loadings are given, and even those aren't complete. Without these reliability and validity numbers, readers have no way to judge whether the three constructs were measured properly.

**4. The tables can't actually be used as they are**

 Tables 1 to 4 are cut off and garbled in places (things like "χ2 ... 402," "TP2 −0.…," "Squ… TP1"). This is probably just a formatting problem from how the document was converted, rather than what was actually submitted. But as it stands, I can't verify the numbers being reported. For example, there's a negative loading on TP2 mentioned in Table 4 that's never discussed anywhere in the text, and a negative loading like that is usually a red flag that needs an explanation or should lead to the item being removed.

**5. Literacy is measured through teachers, not through the children themselves**

 The children's science literacy and environmental literacy are both measured using questionnaires filled in by teachers, not by directly testing or observing the children. This is a real limitation that isn't clearly flagged: the paper is really measuring what teachers *think* about the children's literacy, and the same teachers are also the ones rating PIL-SMART. That means the PIL-SMART → literacy relationships could be affected by common method bias, since it's all coming from one source using one instrument. This should be openly acknowledged as a limitation, and ideally tested statistically (for example, with a Harman's single-factor test).

**6. The sample size may be too small for how complex the model is**

 200 people were surveyed for a model with 3 constructs, each with 10 indicators (30 observed variables total). This is on the low end for typical SEM sample-size guidance, most rules of thumb suggest 10 to 20 respondents per parameter, and this model likely has more parameters than that ratio comfortably allows. The paper says the sample "meets minimum recommendations" but doesn't show the actual parameter count or say which SEM sample-size rule it's using. This claim needs to be backed up with a real calculation and a citation.

**7. The Discussion repeats itself too much**

 Several paragraphs say the same things over and over, that exploratory learning improves science literacy, that teacher perception matters, that environmental literacy needs sustained experience, just reworded slightly each time. For example, the point about teacher perception affecting implementation quality appears almost word-for-word in the Introduction, Discussion paragraph 3, and Discussion paragraph 9. Tightening this up would make the paper much stronger, right now it has less substance per page than the findings deserve, and reviewers are likely to flag this.

**8. Some citations don't match the actual claim being made**

 A few citations feel generic rather than tied to the specific point being made, for example, attributing an idea to "Piaget's view" but citing (Lev et al., 2020; Siraj-Blatchford, 2016) instead of Piaget's own work. Dewey and Bruner are named directly too, but cited through (Nimmo & Park, 2009; Wight et al., 2016) rather than their original writing. This is common in some publishing traditions, but a strict reviewer may ask for more direct engagement with the original theorists.

**9. The consent process needs a stronger justification**

 The study used verbal (not written) informed consent, justified as "minimal risk" with no "sensitive personal data" collected. That's plausible for a teacher survey, but the justification should point to a specific ethical guideline or institutional policy that allows verbal consent for minimal-risk research, rather than just the researchers' own judgement of the risk level.

**10. The generalisability claim in the Limitations needs to be more specific**

 The Limitations section says the results "may not be able to be generalised widely" because of the sample coming from "a number of respondents from certain early childhood education institutions," but it doesn't say which regions or provinces of Indonesia were involved. This matters, since early childhood education quality and resourcing (PAUD) is known to vary a lot across different parts of Indonesia.

**Smaller editorial issues**

- The title uses "Semlj" in one place and "SEMLj" elsewhere, this needs to be made consistent throughout, including in the title itself.
- Several sentences switch awkwardly between passive and active voice (for example, "This condition has an impact on the lack of development..." could be tightened).
- The Abstract says the model shows "excellent match" but doesn't mention the fit concern I raised above, abstracts should reflect any caveats present in the full paper.
- H1 to H3 are stated but never formally confirmed or rejected in the Results using clear language like "H1 is supported" or "H1 is not supported." Adding this would help readers scanning for the outcomes.

**In summary, major revisions are required: **I want to be clear about this: the underlying research question, looking at interdisciplinary early childhood pedagogy, teacher perception as a factor, and whether science literacy feeds into environmental literacy, genuinely excites me, and it's a worthwhile, under-explored contribution. But the statistical reporting needs to be consistent and complete before I can trust the paper's conclusions at face value.

**Declaration on the use of AI in this review**

 I used an AI tool (Claude, Anthropic) to help organise and articulate my assessment of this manuscript. I identified the statistical, conceptual, and presentational issues myself through my own reading of the paper, and I directed the AI to help me rewrite my observations in plain, clear language. All substantive judgements, conclusions, and the overall recommendation in this review are my own.

Is the work clearly and accurately presented and does it cite the current literature?

Partly

If applicable, is the statistical analysis and its interpretation appropriate?

No

Are all the source data underlying the results available to ensure full reproducibility?

No

Is the study design appropriate and is the work technically sound?

Partly

Are the conclusions drawn adequately supported by the results?

No

Are sufficient details of methods and analysis provided to allow replication by others?

No

Reviewer Expertise:

Innovative early childhood STEM education

I confirm that I have read this submission and believe that I have an appropriate level of expertise to confirm that it is of an acceptable scientific standard, however I have significant reservations, as outlined above.

# Response

**Published:** 2026-08-04

**Authors:** Nita Priyanti

**Affiliations:** Early Childhood Education, Universitas Panca Sakti Bekasi, Bekasi, West Java, Indonesia

We sincerely thank the reviewer for the careful, constructive, and detailed assessment of our manuscript. All comments and recommendations have been carefully considered and addressed in the revised version.

 We re-examined the original data, corrected the coding of negatively worded items, repeated the reliability, measurement-model, and structural analyses, and revised all statistical interpretations. The updated model now reports consistent positive coefficients, realistic model-fit indices, complete standardized loadings, Cronbach’s alpha, McDonald’s omega, Composite Reliability, Average Variance Extracted, HTMT, and a comparison between the proposed three-construct model and an alternative single-factor model.

 The tables and figures have also been completely reconstructed to ensure that all results are legible and verifiable.

 We clarified that science and environmental literacy were measured through teachers’ ratings rather than direct assessments of children and expanded the discussion of common method bias, sample size, cross-sectional design, generalizability, and other limitations. The study location, participant criteria, number of estimated parameters, ethical approval, verbal consent procedure, and data-analysis procedures have been described more explicitly. The Discussion was condensed to remove repetition, and citations, theoretical attributions, references, terminology, and language were reviewed for consistency.

 Finally, the Abstract, Results, Discussion, and Conclusion were rewritten to reflect the updated findings without making unsupported causal claims. H1, H2, and H3 are now formally reported as supported based on the revised analysis. We greatly appreciate the reviewer’s comments, which substantially improved the methodological transparency, statistical accuracy, clarity, and overall quality of the manuscript.

# Reviewer report: Reviewer response for version 1

**DOI:** 10.5256/f1000research.200894.r501326

**Published:** 2026-07-20

**Authors:** Husamah H

**Affiliations:** 1 University of Muhammadiyah, Malang East, Java, Indonesia

1. The introduction doesn't explicitly outline the solutions previous researchers have taken to address the problem at hand. The author shouldn't jump straight to the idea, but rather should clearly identify the gaps and justify the urgency of this idea.

 2. The author needs to be consistent in writing: PIL-SMART or Pil-Smart? The PIL-SMART (Project, Inquiry, Science, Mathematics, Art, Religion, and Technology) model, where you don't mention the abbreviation for the "L," or Project Inquiry Learning Science, Mathematics, Art, Religion, and Technology (PIL-SMART)? The reference information for PIL-SMART is very weak! Is this a new model? Who developed it? There should be citations. The inclusion of citations in mentioning this model is ambiguous.

 3. Aspects of research limitations have not been included in the discussion.

 4. Reference writing remains inconsistent. Many references have titles in all capital letters.

Is the work clearly and accurately presented and does it cite the current literature?

Partly

If applicable, is the statistical analysis and its interpretation appropriate?

Yes

Are all the source data underlying the results available to ensure full reproducibility?

Yes

Is the study design appropriate and is the work technically sound?

Yes

Are the conclusions drawn adequately supported by the results?

Yes

Are sufficient details of methods and analysis provided to allow replication by others?

Yes

Reviewer Expertise:

Environmental Literacy, Ecology, And Sustainability Science

I confirm that I have read this submission and believe that I have an appropriate level of expertise to confirm that it is of an acceptable scientific standard, however I have significant reservations, as outlined above.

# Response

**Published:** 2026-07-20

**Authors:** Nita Priyanti

**Affiliations:** Early Childhood Education, Universitas Panca Sakti Bekasi, Bekasi, West Java, Indonesia

Thank you very much for your valuable comments and constructive suggestions. We sincerely appreciate the time and effort you have devoted to reviewing our manuscript. Your feedback is highly valuable and has helped us improve the quality of our work.

 We have carefully considered all of your comments and have revised the manuscript accordingly. Each of the issues raised has been addressed in the revised version, including improvements to the introduction, clarification and consistent presentation of the PIL-SMART model, the addition of the study limitations, and the standardization of the references in accordance with the journal's guidelines.

 We believe that these revisions have significantly strengthened the manuscript and hope that the revised version meets your expectations.

 Thank you once again for your thoughtful review and valuable suggestions.
